# Artificial intelligence in digital pathology: a systematic review and meta-analysis of diagnostic test accuracy

**DOI:** 10.1038/s41746-024-01106-8

**Published:** 2024-05-04

**Authors:** Clare McGenity, Emily L. Clarke, Charlotte Jennings, Gillian Matthews, Caroline Cartlidge, Henschel Freduah-Agyemang, Deborah D. Stocken, Darren Treanor

**Affiliations:** 1 University of Leeds, Leeds, UK; 2 Leeds Teaching Hospitals NHS Trust, Leeds, UK; 3 Department of Clinical Pathology and Department of Clinical and Experimental Medicine, Linköping University, Linköping, Sweden; 4 Centre for Medical Image Science and Visualization (CMIV), Linköping University, Linköping, Sweden

**Keywords:** Medical imaging, Pathogenesis

## Abstract

Ensuring diagnostic performance of artificial intelligence (AI) before introduction into clinical practice is essential. Growing numbers of studies using AI for digital pathology have been reported over recent years. The aim of this work is to examine the diagnostic accuracy of AI in digital pathology images for any disease. This systematic review and meta-analysis included diagnostic accuracy studies using any type of AI applied to whole slide images (WSIs) for any disease. The reference standard was diagnosis by histopathological assessment and/or immunohistochemistry. Searches were conducted in PubMed, EMBASE and CENTRAL in June 2022. Risk of bias and concerns of applicability were assessed using the QUADAS-2 tool. Data extraction was conducted by two investigators and meta-analysis was performed using a bivariate random effects model, with additional subgroup analyses also performed. Of 2976 identified studies, 100 were included in the review and 48 in the meta-analysis. Studies were from a range of countries, including over 152,000 whole slide images (WSIs), representing many diseases. These studies reported a mean sensitivity of 96.3% (CI 94.1–97.7) and mean specificity of 93.3% (CI 90.5–95.4). There was heterogeneity in study design and 99% of studies identified for inclusion had at least one area at high or unclear risk of bias or applicability concerns. Details on selection of cases, division of model development and validation data and raw performance data were frequently ambiguous or missing. AI is reported as having high diagnostic accuracy in the reported areas but requires more rigorous evaluation of its performance.

## Introduction

**Fig. 1:**
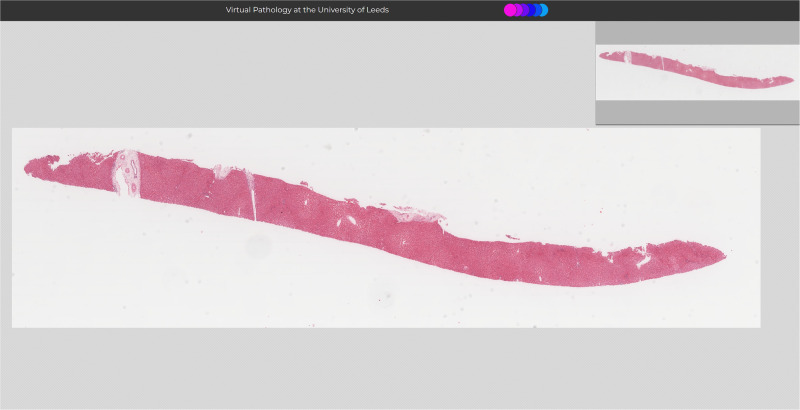
Example whole slide image (WSI) of a liver biopsy specimen at low magnification. These are high resolution digital pathology images viewed by a pathologist on a computer to make a diagnostic assessment. Image from www.virtualpathology.leeds.ac.uk^143^.

Following recent prominent discoveries in deep learning techniques, wider artificial intelligence (AI) applications have emerged for many sectors, including in healthcare^1–3^. Pathology AI is of broad importance in areas across medicine, with implications not only in diagnostics, but in cancer research, clinical trials and AI-enabled therapeutic targeting^4^. Access to digital pathology through scanning of whole slide images (WSIs) has facilitated greater interest in AI that can be applied to these images^5^. WSIs are created by scanning glass microscope slides to produce a high resolution digital image (Fig. 1), which is later reviewed by a pathologist to determine the diagnosis^6^. Opportunities for pathologists have arisen from this technology, including remote and flexible working, obtaining second opinions, easier collaboration and training, and applications in research, such as AI^5,6^.

Application of AI to an array of diagnostic tasks using WSIs has rapidly expanded in recent years^5–8^. Successes in AI for digital pathology can be found for many disease types, but particularly in examples applied to cancer^4,9–11^. An important early study in 2017 by Bejnordi et al. described 32 AI models developed for breast cancer detection in lymph nodes through the CAMELYON16 grand challenge. The best model achieved an area under the curve (AUC) of 0.994 (95% CI 0.983–0.999), demonstrating similar performance to the human in this controlled environment^12^. A study by Lu et al. in 2021 trained AI to predict tumour origin in cases of cancer of unknown primary (CUP)^13^. Their model achieved an AUC of 0.8 and 0.93 for top-1 and top-3 tumour accuracies respectively on an external test set. AI has also been applied to making predictions, such as determining the 5-year survival in colorectal cancer patients and the mutation status across multiple tumour types^14,15^.

Several reviews have examined the performance of AI in subspecialties of pathology. In 2020, Thakur et al. identified 30 studies of colorectal cancer for review with some demonstrating high diagnostic accuracy, although the overall scale of studies was small and limited in their clinical application^16^. Similarly in breast cancer, Krithiga et al. examined studies where image analysis techniques were used to detect, segment and classify disease, with reported accuracies ranging from 77 to 98%^17^. Other reviews have examined applications in liver pathology, skin pathology and kidney pathology with evidence of high diagnostic accuracy from some AI models^18–20^. Additionally, Rodriguez et al. performed a broader review of AI applied to WSIs and identified 26 studies for inclusion with a focus on slide level diagnosis^21^. They found substantial heterogeneity in the way performance metrics were presented and limitations in the ground truth used within studies. However, their study did not address other units of analysis and no meta-analysis was performed. Therefore, the present study is the first systematic review and meta-analysis to address the diagnostic accuracy of AI across all disease areas in digital pathology, and includes studies with multiple units of analysis.

Despite the many developments in pathology AI, examples of routine clinical use of these technologies remain rare and there are concerns around the performance, evidence quality and risk of bias for medical AI studies in general^22–24^. Although, in the face of an increasing pathology workforce crisis, the prospect of tools that can assist and automate tasks is appealing^25,26^. Challenging workflows and long waiting lists mean that substantial patient benefit could be realised if AI was successfully harnessed to assist in the pathology laboratory.

This systematic review provides an overview of performance of diagnostic tools across histopathology. The objective of this review was to determine the diagnostic test accuracy of artificial intelligence solutions applied to WSIs to diagnose disease. A further objective was to examine the risk of bias and applicability concerns within the papers. The aim of this was to provide context in terms of bias when examining the performance of different AI tools (Fig. 1).

## Results

### Study selection

**Fig. 2:**
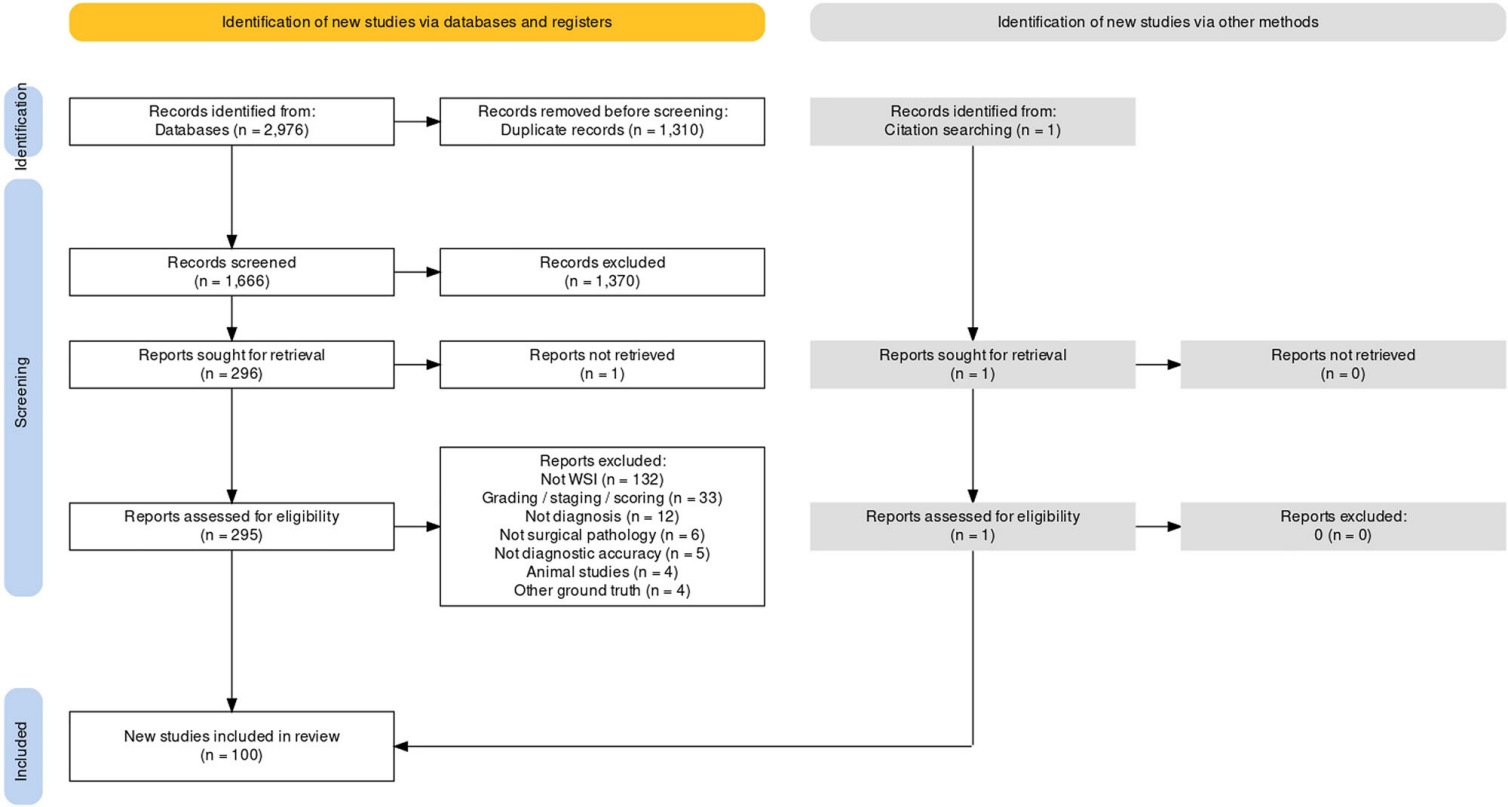
Study selection flow diagram. Generated using PRISMA2020 at https://estech.shinyapps.io/prisma_flowdiagram/^144^.

Searches identified 2976 abstracts, of which 1666 were screened after duplicates were removed. 296 full text papers were reviewed for potential inclusion. 100 studies met the full inclusion criteria for inclusion in the review, with 48 studies included in the full meta-analysis (Fig. 2*)*.

### Study characteristics

Study characteristics are presented by pathological subspecialty for all 100 studies identified for inclusion in Tables 1–7. Studies from Europe, Asia, Africa, North America, South America and Australia/Oceania were all represented within the review, with the largest numbers of studies coming from the USA and China. Total numbers of images used across the datasets equated to over 152,000 WSIs. Further details, including funding sources for the studies can be found in Supplementary table 10. Tables 1 and 2 show characteristics for breast pathology and cardiothoracic pathology studies respectively. Tables 3 and 4 are characteristics for dermatopathology and hepatobiliary pathology studies respectively. Tables 5 and 6 have characteristics for gastrointestinal and urological pathology studies respectively. Finally, Table 7 outlines characteristics for studies with multiple pathologies examined together and for other pathologies such as gynaepathology, haematopathology, head and neck pathology, neuropathology, paediatric pathology, bone pathology and soft tissue pathology.

**Table 1:** Characteristics of breast pathology studies

First author, year & reference	Location	Index test	Disease studied	Reference standard	Data sources	Training set details	Validation set details	Test set details	External validation	Unit of analysis
Cengiz^47^	Turkey	CNN	Breast cancer	Not stated	Not stated	296,675 patches		101,706 patches	Unclear	Patch/Tile
Choudhary^46^	India, USA	CNN (VGG19, ResNet54, ResNet50)	Breast cancer	Pathologist annotations, slide diagnoses	IDC dataset	194,266 patches		83,258 patches	No	Patch/Tile
Cruz-Roa^94^	Colombia, USA	FCN (HASHI)	Breast cancer	Pathologist annotations	Hospital of the University of Pennsylvania; University Hospitals Case Medical Centre/Case Western Reserve University; Cancer Institute of New Jersey; TCGA	698 cases	52 cases	195 cases	Yes	Pixel
Cruz-Roa^95^	Colombia, USA	CNN (ConvNet)	Breast cancer	Pathologist annotations	University of Pennsylvania Hospital; University Hospitals Case Medical Centre/Case Western Reserve University; Cancer Institute of New Jersey; TCGA	349 patients	40 patients	216 patients	Yes	Pixel
Hameed^45^	Spain, Columbia	CNN (ensemble of fine-tuned VGG16 & fine-tuned VGG19)	Breast cancer	Pathologist labels & annotations	Colsanitas Colombia University	540 images/patches	135 images/patches	170 images/patches	No	Patch/Tile
Jin^44^	Canada	U-net CNN (ConcatNet)	Breast cancer	Labels	PatchCamelyon dataset; Open-source dataset from PMID 27563488; Warwick dataset	262,144 patches + 538 images	32,768 patches	32,768 patches	No	Patch/Tile
Johny^96^	India	Custom deep CNN	Breast cancer	Pathologist patch labels	PatchCamelyon Dataset	262,144 patches		65,536 patches	No	Patch/Tile
Kanavati^43^	Japan	CNN tile classifier (EfficientNetB1) + RNN tile aggregator	Breast cancer	Diagnostic review by pathologists	International University of Health and Welfare, Mita Hospital; Sapporo-Kosei General Hospital.	1652 WSIs	90 WSIs	1930 WSIs	Yes	Slide
Khalil^97^	Taiwan	Modified FCN	Breast cancer	Pathologist annotations, IHC.	National Taiwan University Hospital dataset	68 WSIs		26 WSIs	No	Slide
Lin^98^	Hong Kong, China, UK	Modified FCN	Breast cancer	Slide level labels, pathologist annotations	Camelyon dataset	202 WSIs	68 WSIs	130 WSIs	No	Slide
Roy^99^	India, Germany	Multiple machine learning classifiers (CatBoost & others)	Breast cancer	Unclear	IDC Breast Histopathology Image Dataset	Unclear	Unclear	Unclear	No	Patch/Tile
Sadeghi^100^	Germany, Austria	CNN	Breast cancer	Pathologist supervised annotations, IHC	Camelyon17 dataset; Camelyon16 dataset	400 WSIs	100 WSIs	20,000 patches	No	Patch/Tile
Steiner^101^	USA	CNN (LYNA - Inception framework)	Breast cancer	Pathologist review, IHC	Camelyon; Expired clinical archive blocks from 2 sources	215 WSIs	54 WSIs	70 WSIs	Yes	Slide
Valkonen^102^	Finland	Random forest	Breast cancer	Pathologist WSI annotations	Camelyon16 dataset	1,000,000 patches	270 WSIs leave-one-out cross validation		Yes	Patch/Tile
Wang Q^42^	China	SoMIL) + adaptive aggregator + RNN	Breast cancer	WSI labels, pixel level annotations of metastases	Camelyon16; MSK breast cancer metastases dataset	289 WSIs		240 WSIs	Yes	Slide
Wu^41^	USA	ROI classifier + Tissue segmentation CNN +;Diagnosis classifier SVM	Breast cancer	Pathologist pixel labels	Breast Cancer Surveillance Consortium–associated tumour registries in New Hampshire and Vermont	58 ROIs	Cross validation 428 ROIs		Unclear	Other (ROIs)

**Table 2:** Characteristics of cardiothoracic pathology studies

First author, year & reference	Location	Index test	Disease studied	Reference standard	Data sources	Training set details	Validation set details	Test set details	External validation	Unit of analysis
Chen^103^	Taiwan	CNN	Lung cancer	Pathologist diagnosis,slide level labels.	Taipei Medical University Hospital; Taipei Muncipal Wanfang Hospital; Taipei Medical University Shuang-Ho Hospital; TCGA.	5045 WSIs	561 WSIs	2441 WSIs	Yes	Slide
Chen^104^	China	CNN (EfficientNetB5)	Lung cancer	Pathologist annotations	Hospital of Sun Yat-sen University; Shenzhen People’s Hospital; Cancer Centre of Guangzhou Medical University		813 cases train & validate	1101 cases	Yes	Slide
Coudray^105^	USA, Greece	CNN (Inception v3)	Lung cancer	Pathologist labels	TCGA, New York University	1157 WSIs	234 WSIs	584 WSIs	Yes	Slide
Dehkharghanian^106^	Canada, USA	DNN (KimiaNet)	Lung cancer	WSI diagnostic label	TCGA; Grand River Hospital, Kitchener, Canada.	575 WSIs	79 WSIs	81 WSIs	Yes	Patch/Tile
Kanavati^68^	Japan	CNN (EfficientNet-B3)	Lung cancer	Pathologist review & annotations	Kyushu Medical Centre; Mita Hospital; TCGA; TCIA	3554 WSIs	150 WSIs	2170 WSIs	Yes	Slide
Wang X^57^	China, Hong Kong, UK	FCN + Random Forest classifier	Lung cancer	Pathologist annotations, WSI labels.	Sun Yat-sen University Cancer Centre (SUCC); TCGA	1154 WSIs		285 WSIs	Yes	Slide
Wei^107^	USA	CNN (ResNet)	Lung cancer	Pathologist WSI labels	Dartmouth-Hitchcock Medical Centre (DHMC)	245 WSIs	34 WSIs	143 WSIs	No	Slide
Yang^108^	China	CNN (EfficientNetB5; ResNet50)	Lung cancer	Pathologist diagnosis, IHC, medical records.	Sun Yat-sen University; Shenzhen People’s Hospital; TCGA	511 WSIs	115 WSIs	1067 WSIs	Yes	Patch/Tile
Zhao^55^	China	Combined (MR-EM-CNN + HMS + RNN + RMDL)	Lung cancer	Pathologist annotations, patch labels.	TCGA	1481 WSIs	321 WSIs	323 WSIs	No	Slide
Zheng^109^	USA	CNN (GTP: Graph transformer + node representation connectivity information + feature generation & contrastive learning)	Lung cancer	Pathologist annotations, WSI level labels.	Clinical Proteomic Tumour Analysis Consortium (CPTAC), TCGA; the National Lung Screening Trial (NLST)		2071 WSIs 5 fold cross validation	2082 WSIs	Yes	Slide
Uegami^110^	Japan	CNN (ResNet18) + K means clustering + pathologist clustering + transfer learning	Interstitial lung disease	Pathologist diagnosis	1 institute (unclear)	126 cases	54 cases	180 WSIs (51 cases)	No	Patch/Tile

**Table 3:** Characteristics of dermatopathology studies

First author, year & reference	Location	Index test	Disease studied	Reference standard	Data sources	Training set details	Validation set details	Test set details	External validation	Unit of analysis
Kimeswenger^111^	Austria, Switzerland	CNN + ANN (Feature constructor ImageNet CNN + classification ANN)	Basal cell carcinoma	Categorised by pathologist	Kepler University Hospital; Medical University of Vienna.	688 WSIs		132 WSIs	No	Patch/Tile
Alheejawi^87^	Canada, India	CNN	Melanoma	MART-1 stained images	University of Alberta, Canada	70,960 × 960 pixel images	15,960 × 960 pixel images	15 960 × 960 pixel images	No	Pixel
De Logu^72^	Italy	CNN (Inception ResNet v2)	Melanoma	Pathologist review	University of Florence; University Hospital of Siena; Institute of Biomolecular Chemistry, National research Council	45 WSIs	15 WSIs	40 WSIs	No	Patch/Tile
Hekler^70^	Germany	CNN (ResNet50)	Melanoma	Image labels	Dr Dieter Krahl institute, Heidelberg	595 cropped images		100 cropped images	No	Patch/Tile
Hohn^69^	Germany	CNN (ResNeXt50)	Melanoma	Pathologist diagnosis	Two laboratories unspecified	232 WSIs	67 WSIs	132 WSIs	No	Slide
Li^112^	China	CNN (ResNet50)	Melanoma	Pathologist WSI annotations	Central South University Xiangya Hospital; TCGA	491 WSIs	105 WSIs	105 WSIs	No	Slide
Wang L^58^	China	CNN for patch-level classification (VGG16) & random forest for WSI-level classification	Melanoma	Pathologist diagnosis, consensus, IHC, annotations.	Zhejiang University School of Medicine; Ninth People’s Hospital of Shanghai	105,415 patches	1962 patches	118,123 patches	Yes	Patch/Tile
del Amor^113^	Spain	CNN (VGG16, ResNet50, InceptionV3, MobileNetV2)	Spitzoid skin tumours	Pathologist annotations	CLARIFYv1	36 WSIs	5 fold cross validation of training set	15 WSIs	No	Unclear

**Table 4:** Characteristics of hepatobiliary pathology studies

First author, year & reference	Location	Index test	Disease studied	Reference standard	Data sources	Training set details	Validation set details	Test set details	External validation	Unit of analysis
Aatresh^74^	India	CNN (LiverNet)	Liver cancer	Pathologist annotations	Kasturba Medical College (KMC); TCGA	5 fold cross-validation 5450 samples			No	Patch/Tile
Chen^114^	China	CNN (Inception V3)	Liver cancer	Labels	TCGA, Sir Run-Run Shaw Hospital	278 WSIs	56 WSIs	258 WSIs	Yes	Patch/Tile
Kiani^115^	USA	CNN (Densenet)	Liver cancer	Pathologist diagnosis, consensus, IHC, special stains	TCGA; Stanford whole-slide image dataset	20 WSIs	50 WSIs	106 WSIs	Yes	Slide
Yang^116^	Taiwan	Feature Aligned Multi-Scale Convolutional Network (FA-MSCN)	Liver cancer	Pathologist labels and ROIs	Unclear	20 WSIs		26 WSIs	Unclear	Unclear
Schau^62^	USA, Thailand	CNNs (Inception v4)	Liver metastases	Pathologist labels, annotations	OHSU Knight BioLibrary	200 WSIs		85 WSIs	No	Patch/Tile
Fu^71^	China	CNN (InceptionV3 patch-level classification), lightGBM model (WSI-level classification) & U-Net CNN (patch-level segmentation)	Pancreatic cancer	Pathologist annotations, labels	Peking Union Medical College Hospital (PUMCH); TCGA	79,588 patches	9952 patches	9948 patches +52 WSIs	Yes	Slide
Naito^63^	Japan	CNN (EfficientNetB1)	Pancreatic cancer	Pathologist review, pathologist annotations	Kurume University	372 WSIs	40 WSIs	120 WSIs	No	Slide
Song^60^	South Korea	Bayesian classifier; k-NN; SVM; ANN.	Pancreatic neoplasms	Unclear	Pathology department of Yeognam University	240 patches		160 patches	No	Patch/Tile

**Table 5:** Characteristics of gastrointestinal studies

First author, year & reference	Location	Index test	Disease studied	Reference standard	Data sources	Training set details	Validation set details	Test set details	External validation	Unit of analysis
Sali^117^	USA	CNN & Random forest; SVM; k-means; GMM	Barrett’s Oesophagus	Pathologist consensus, pixel-wise annotations	Hunter Holmes McGuire Veterans Affairs Medical Centre	115 WSIs	535 WSIs 10 fold cross validation		No	Slide
Syed^118^	USA, Pakistan, Zambia, UK	CNN (ResNet50; ResNet50 multi-zoom; shallow CNN; ensemble).	Coeliac & Environmental Enteropathathy	Slide level diagnosis, IHC, patch labels.	Aga Khan University; University of Zambia & University Teaching Hospital Zambia; University of Virginia, USA	231 WSIs	115 WSIs	115 WSIs	Unclear	Slide
Nasir-Moin^119^	USA	CNN (ResNet18)	Colorectal adenoma/polyps	Pathologist consensus	Dartmouth-Hitchcock Medical Centre (DHMC). Prior validation on 24 US institutions	508 WSIs		100 WSIs + Previous validation 238 WSIs	Yes	Slide
Song^36^	China	CNN (DeepLab v2 + ResNet34)	Colorectal adenoma/polyps	Pathologist labels	Chinese People’s Liberation Army General Hospital (PLAGH); China-Japan Friendship Hospital (CJFH); Cancer Hospital, Chinese Academy of Medical Science (CH).	177 WSIs	40 WSIs	362 WSIs	Yes	Slide
Wei^120^	USA	CNN (ResNet)	Colorectal adenoma/polyps	Pathologist annotations	Dartmouth-Hitchcock Medical Centre (DHMC); External set multiple institutions	326 WSIs	25 WSIs	395 WSIs	Yes	Slide
Feng^121^	China, USA, South Korea	CNN (ensemble of 8 networksmodified U-Net + VGG-16 or VGG-19)	Colorectal cancer	Pixel annotations, pathologist labels	DigestPath 2019 Challenge (task 2)	750 WSIs		250 WSIs	No	Unclear
Haryanto^122^	Indonesia	Conditional Sliding Window (CSW) algorithm used to generate images for CNN 7-5-7	Colorectal cancer	Pathologist labels & annotations	Warwick dataset; University of Indonesia	Unclear	Unclear	Unclear	Unclear	Unclear
Sabol^123^	Slovakia, Japan	CNN + X-CFCMC	Colorectal cancer	Annotations	Publicly available dataset from Kather et al.		10 fold cross validation 5000 tiles		No	Patch/Tile
Schrammen^124^	Germany, Netherlands, UK	Single neural network (SLAM - based on ShuffleNet)	Colorectal cancer	Patient/slide level labels	DACHS study, YCR-BCIP	2448 cases		889 cases	Yes	Slide
Tsuneki^34^	Japan	CNN (EfficientNetB1)	Colorectal cancer	Pathologist diagnosis & annotations	Wajiro, Shinmizumaki, Shinkomonji, & Shinyukuhashi hospitals, Fukuoka; Mita Hospital, Tokyo	680 WSIs	68 WSIs	1799 WSIs	Yes	Slide
Wang KS^40^	China, USA	CNN (Inception V3)	Colorectal cancer	Pathologist consensus & labels	14 hospitals/sources	559 WSIs	283 WSIs	At least 13,838 WSIs	Yes	Patch/Tile
Wang C^32^	China	CNN (bilinear)	Colorectal cancer	Annotations	University Medical Centre Mannheim, Heidelberg		5 fold cross validation on 1000 patches		No	Patch/Tile
Xu^30^	China	Dual resolution deep learning network with self-attention mechanism (DRSANet)	Colorectal cancer	Pathologist annotations, Patch labels, Pathologist pixel annotations.	TCGA; Affiliated Cancer Hospital and Institute of Guangzhou Medical University (ACHIGMU)	100,000 patches	40,000 patches	80,000 patches	Yes	Patch/Tile
Zhou^125^	China, Singapore	CNN (ResNet) + Random Forest	Colorectal cancer	Pathologist slide labels, reports, annotations & consensus	TGCA; Hospital of Zhejiang University; Hospital of Soochow University; Nanjing First Hospital	950 WSIs		446 WSIs	Yes	Slide
Ashraf^39^	South Korea	CNN (DenseNet-201)	Gastric cancer	Pathologist review & annotations	Seegene Medical Foundation in South Korea; Camelyon	Primary model: 723 WSIs; LN model: 262,11 patches	Primary model: 91 WSIs; LN model: 32,768 patches	Primary model: 91 WSIs; LN model: 32,768 patches	No	Patch
Cho^38^	South Korea	CNN (AlexNet; ResNet50; Inception-v3)	Gastric cancer	Labels	TCGA-STAD; SSMH Seoul St. Mary’s Hospital dataset		10 fold cross validation		Yes	Slide
Ma^126^	China	CNN (modified InceptionV3) + random forest classifier	Gastric cancer	Pathologist annotations	Ruijin Hospital	534 WSIs	76 WSIs	153 WSIs	No	Slide
Rasmussen^37^	Canada	CNN (DenseNet169)	Gastric cancer	Pathologist annotations	Queen Elizabeth II Health Sciences Centre & Dalhousie University; Sunnybrook Health Science Centre, University of Toronto	14,266 patches	1585 patches	1785 patches	Yes	Patch/Tile
Song^85^	China, USA	CNN (Multiple models); random forest	Gastric cancer	Pathologist pixel level annotations	PLAGH dataset; Multicentre dataset (PUMCH, CHCAMS & Pekin Union Medical College)	2860 WSIs	300 WSIs	4993 WSIs	Yes	Slide
Tung^33^	Taiwan	CNN (YOLOv4)	Gastric cancer	Pathologist annotations	Taiwan Cancer Registry Database	2200 image tiles		550 image tiles	No	Patch/Tile
Wang S^31^	China	Recalibrated multi-instance deep learning method (RMDL)	Gastric cancer	Pathologist pixel annotations	Sun Yat-sen University	408 WSIs		200 WSIs	No	Slide
Ba^127^	China	CNN (ResNet50)	Gastritis	Pathologist review & pixel annotations	Chinese People’s Liberation Army General Hospital	1008 WSIs	100 WSIs	142 WSIs	No	Slide
Steinbuss^35^	Germany	CNN (Xception)	Gastritis	Diagnoses – modified Sydney Classification, pathologist annotations	Institute of Pathology, University Clinic Heidelberg	825 patches	196 patches	209 patches	No	Patch/Tile
Iizuka^28^	Japan	CNN (InceptionV3 + max-pooling or RNN aggregator)	Multiple (Colorectal cancer & Gastric tumours)	Pathologist annotations	Hiroshima University Hospital dataset; Haradoi Hospital dataset; TCGA dataset	Stomach: 3628 WSIs; Colon: 3536 WSIs		Stomach: 1475 WSIs; Colon: 1574 WSIs	Yes	Slide

**Table 6:** Characteristics of urological pathology studies

First author, year & reference	Location	Index test	Disease studied	Reference standard	Data sources	Training set details	Validation set details	Test set details	External validation	Unit of analysis
da Silva^54^	Brazil, USA	CNN (Paige Prostate 1.0)	Prostate cancer	Pathologist consensus, IHC	Instituto Mario Penna, Brazil	Prior study: trained on 2000 WSIs		661 WSIs (579 part specimens)	Yes	Other (part specimen level)
Duran-Lopez^128^	Spain	CNN (PROMETEO) + Wide and deep neural network	Prostate cancer	Pathologist pixel annotations	Pathological Anatomy Unit of Virgen de Valme Hospital, Spain		5 fold cross validation	332 WSIs	No	Slide
Esteban^53^	Spain	Optical density granulometry-based descriptor + Gaussian processes	Prostate cancer	Pathologist pixel annotations	SICAPv1 database; Prostate cancer database by Gertych et al.		60 WSIs 5 fold cross validation	19 WSIs + 593 patches	Yes	Patch/Tile
Han^129^	Canada	Multiple ML approaches (Transfer learning with TCMs & others)	Prostate cancer	Pathologist annotations & supervision	Western University		286 WSIs cross validation for train/test (leave one out)	13 WSIs	No	Patch/Tile
Han^51^	Canada	Traditional ML and 14 texture features extracted from TCMs; Transfer learning with pretrained AlexNet fine-tuned by TCM ROIs; Transfer learning with pretrained AlexNet fine-tuned with raw image ROIs	Prostate cancer	Pathologist annotations & supervision	Western University		286 WSIs cross validation for train/test (leave one out)	13 WSIs	No	Patch/Tile
Huang^130^	USA	CNN (U-Net gland segmenter) + CNN feature extractor & classifier	Prostate cancer	Pathologist review, patch annotations using ISUP criteria.	University of Wisconsin Health System	838 WSIs		162 WSIs	No	Other (patch-pixel level)
Swiderska-Chadaj^50^	Netherlands, Sweden	CNN (U-Net, DenseNetFCN, EfficientNet)	Prostate cancer	Slide level labels, pathologist annotations	The Penn State Health Department of Pathology; PAMM Laboratorium voor Pathologie; Radboud University Medical Centre.	264 WSIs	60 WSIs	297 WSIs	Yes	Slide
Tsuneki^49^	Japan	Transfer learning (TL-colon poorly ADC-2 (20×, 512)); CNN (EfficientNetB1 20×, 512); CNN (EfficientNetB1 (10×, 224)	Prostate cancer	Pathologist diagnosis & consensus	Wajiro, Shinmizumaki, Shinkomonji, and Shinyukuhashi hospitals, Fukuoka; TGCA	1122 WSIs	60 WSIs	2512 WSIs	Yes	Slide
Abdeltawab^131^	USA, UAE	CNN (pyramidal)	Renal cancer	Pathologist review & annotations	Indiana University, USA	38 WSIs	6 WSIs	20 WSIs	No	Pixel
Fenstermaker^52^	USA	CNN	Renal cancer	Pathology report	TCGA		15,168 patches train/validate	4286 patches	No	Patch/Tile
Tabibu^132^	India	CNNs (ResNet18 & 34) + SVM (DAG-SVM)	Renal cancer	Clinical information including pathology reports	TCGA	1474 WSIs	317 WSIs	314 WSIs	Yes	Slide
Zhu^48^	USA	CNN (ResNet-18) + Decision Tree	Renal cancer	Pathologist annotations	Dartmouth-Hitchcock Medical Centre (DHMC); TCGA	385 WSIs	23 WSIs	1074 WSIs	Yes	Slide

**Table 7:** Characteristics of other pathology/multiple pathology studies

First author, year & reference	Location	Index test	Disease studied	Reference standard	Data sources	Training set details	Validation set details	Test set details	External validation	Unit of analysis
BenTaieb^133^	Canada	K means + LSVM	Ovarian cancer	Pathologist consensus	Not stated	68 WSIs		65 WSIs	No	Slide
Shin^61^	South Korea	CNN (Inception V3)	Ovarian cancer	Pathologist diagnosis	TCGA; Ajou University Medical Centre	7245 patches		3051 patches	Yes	Patch/Tile
Sun^59^	China	CNN (HIENet)	Endometrial cancer	Pathologist consensus, patch labels	2 datasets from Hospital of Zhenghou University		10 fold cross validation on 3300 patches	200 patches	No	Patch/Tile
Yu^134^	USA	CNN (VGGNet, GoogLeNet; AlexNet)	Ovarian cancer	Pathology reports and pathologist review	TCGA	1100 WSIs		275 WSIs	No	Slide
Achi^73^	USA	CNN	Lymphoma	Labels	Virtual pathology at University of Leeds, Virtual Slide Box University of Iowa	1856 patches	464 patches	240 patches	No	Patch/Tile
Miyoshi^65^	Japan, USA	deep neural network classifier with averaging method	Lymphoma	Pathologist annotations, IHC	Kurume University	Unclear	Unclear	100 patches	No	Patch/Tile
Mohlman^64^	USA	deep densely connected CNN	Lymphoma	Unclear - likely slide diagnosis	University of Utah dataset, Mayo Clinic Rochester dataset	8796 patches		2037 patches	No	Patch/Tile
Syrykh^135^	France	CNNs (“Several Deep CNNs” + Bayesian Neural Network)	Lymphoma	Slide diagnosis, IHC, patch labels	Toulouse University Cancer Institute, France; Dijon University Hospital, France.	221 WSIs	111 WSIs	159 WSIs	No	Slide
Yu^136^	USA	CNN (VGGNet & others)	Lymphoma	Pathologist consensus, IHC	TCGA & International Cancer Genome Consortium (ICGC)	707 patients		302 patients	Yes	Patch/Tile
Yu^137^	Taiwan	HTC-RCNN (ResNet50). Decision-tree-based machine learning algorithm, XGBoost	Lymphoma	Pathologist diagnosis with WHO criteria, pathologist annotations	17 hospitals in Taiwan (names not specified)	Detect: 27 ROIs. Classify 3 fold validation from 40 WSIs	Detect: 2 ROIs. Classify: 3 fold validation from 40 WSIs	Detect: 3 ROIs. Classify: 3 fold validation from 40 WSIs	Unclear	Slide
Li^66^	China, USA	CNN (Inception V3)	Thyroid neoplasms	Pathologist review	Peking Union Medical College Hospital	279 WSIs	70 WSIs	259 WSIs	No	Slide
Xu^138^	China	CNN (AlexNet) + SVM classifier	Multiple (Brain tumours, colorectal cancer)	MICCAI brain: Labels Colorectal: Pathologist review & image crops	MICCAI 2014 Brain Tumour Digital Pathology Challenge & colon cancer dataset	Brain:80 images ; Colon: 359 cropped images		Brain: 61 images; Colon: 358 cropped images	No	Patch/Tile
DiPalma^139^	USA	CNN (Resnet architecture but trained from scratch)	Multiple (Coeliac, lung cancer, renal cancer)	RCC & Coeliac: Pathologist diagnosis, Lung: pathologist annotations	TCGA, Darmouth-Hitchcock Medical Centre	Coeliac: 5908 tissue pieces; Lung: 239 WSIs, 2083 tissue pieces; Renal: 617 WSIs, 834 tissue pieces.	Coeliac: 1167 tissue pieces;	Coeliac: 25,284 tissue pieces; Lung: 34 WSIs, 305 tissue pieces; Renal: 265 WSIs, 364 tissue pieces.	No	Slide
Litjens^27^	Netherlands	CNN	Multiple (Prostate cancer; Breast cancer)	Pathologist annotations/supervision, pathology reports.	3 datasets from Radboud University Medical Centre	Prostate: 100 WSIs; Breast: 98 WSIs.	Prostate: 50 WSIs; Breast: 33 WSI.	Prostate: 75 WSIs; Breast: 42 WSIs + Consecutive set: 98 WSIs	No	Slide
Menon^140^	India	FCN (ResNet18)	Multiple cancer types	Slide labels	TCGA	6855 WSIs	1958 WSIs	979 WSIs	No	Patch/Tile
Noorbakhsh^88^	USA	CNN (InceptionV3)	Multiple cancer types	Pathologist annotations	TCGA, CPTAC.	19,470 WSIs		10,460 WSIs	Yes	Slide
Yan^29^	China	Contrastive clustering algorithm to train CNN encoder + recursive cluster refinement method	Multiple (colorectal cancer/polyps, breast cancer)	NCT-CRC Patch classification, CAMELYON16 annotations. In-house: pathologist diagnosis	NCT-CRC dataset; Camelyon16 dataset; In-house colon polyp WSI dataset	NCT-CRC 80,000 patches; Camelyon16 80,000 patches;	NCT-CRC 10,000 patches; Camelyon16 10,000 patches.	NCT-CRC + In house polyp dataset: 10,000 patches + 20 patients; CAMELYON16 10,000 patches	Yes	Patch/Tile
Li^67^	China	CNN (GoogleLeNet)	Brain cancer	Diagnosed WSIs	Huashan Hospital, Fudan University	67 WSIs		139 WSIs	No	Patch/Tile
Schilling^141^	Germany	Voting ensemble classifier (logistic regression, SVM, decision tree & random forest)	Hirschsprung’s disease	Pathologist diagnosis against criteria, IHC	Institute of Pathology, Friedrich-Alexander-University Erlangen Nurnberg, Germany	172 WSIs	58 WSIs	77 WSIs	No	Unclear
Mishra^142^	USA	CNN (LeNet & AlexNet)	Osteosarcoma	Manual annotations by senior pathologists.	Unclear	38,400 patches	12,800 patches	12,800 patches	No	Patch/Tile
Zhang^56^	USA	CNN (Inception V3)	Rhabdomyosarcoma	WSIs reviewed and classified by pathologist	Children’s oncology group biobanking study	56 WSIs	12 WSIs	204 WSIs	Unclear	Patch/Tile

### Risk of bias and applicability

**Fig. 3:**
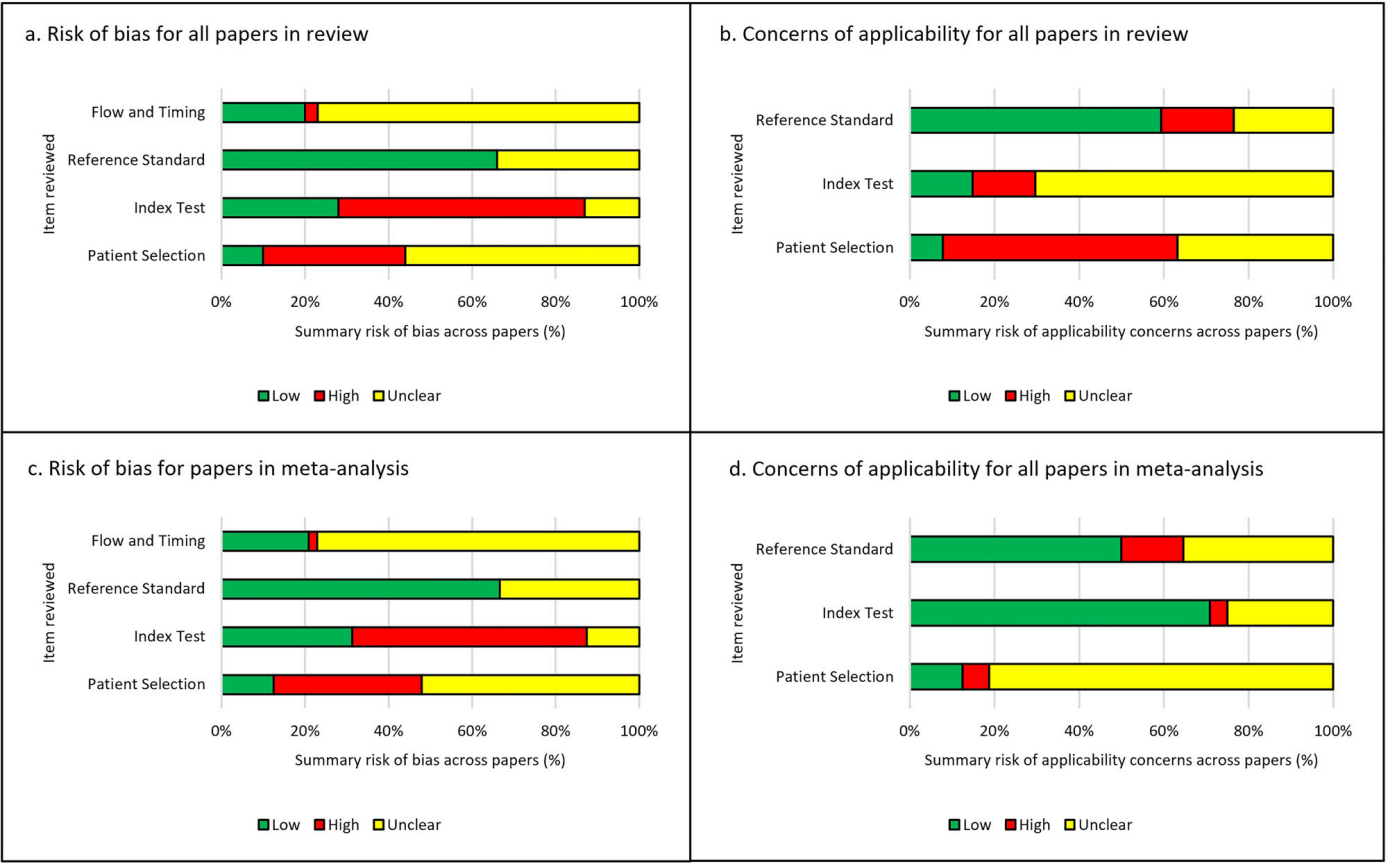
Risk of bias and concerns of applicability in summary percentages for studies included in the review. **a** Summaries for risk of bias for all 100 papers included in the review. **b** Summaries for applicability concerns for all 100 papers included in the review. **c**, **d** Summaries for risk of bias for 48 papers included in the meta-analysis. **d** Summaries for applicability concerns for 48 papers included in the meta-analysis.

The risk of bias and applicability assessment using the tailored QUADAS-2 tool demonstrated that the majority of papers were either at high risk or unclear risk of bias in three out of the four domains (Fig. 3*)*. The full breakdown of individual paper scores can be found in Supplementary Table 1. Of the 100 studies included in the systematic review, 99% demonstrated at least one area at high or unclear risk of bias or applicability concerns, with many having multiple components at risk.

Of the 48 studies included in the meta-analysis (Fig. 3c, d), 47 of 48 studies (98%) were at high or unclear risk of bias or applicability concerns in at least one area examined. 42 of 48 studies (88%) were either at high or unclear risk of bias for patient selection and 33 of 48 studies (69%) were at high or unclear risk of bias concerning the index test. The most common reasons for this included: cases not being selected randomly or consecutively, or the selection method being unclear; the absence of external validation of the study’s findings; and a lack of clarity on whether training and testing data were mixed. 16 of 48 studies (33%) were unclear in terms of their risk of bias for the reference standard, but no studies were considered high risk in this domain. There was often very limited detail describing the reference standard, for example the process for classifying or diagnosing disease, and so it was difficult to assess if this was an appropriate reference standard to use. For flow and timing, to ensure cases were recent enough to the study to be relevant and reasonable quality, one study was at high risk but 37 of 48 studies (77%) were at unclear risk of bias.

There were concerns of applicability for many papers included in the meta-analysis with 42 of 48 studies (88%) with either unclear or high concerns for applicability in the patient selection, 14 of 48 studies (29%) with unclear or high concern for the index test and 24 of 48 studies (50%) with unclear or high concern for the reference standard. Examples for this included; ambiguity around the selection of cases and the risk of excluding subgroups, and limited or no details given around the diagnostic criteria and pathologist involvement when describing the ground truth.

### Synthesis of results

**Fig. 4:**
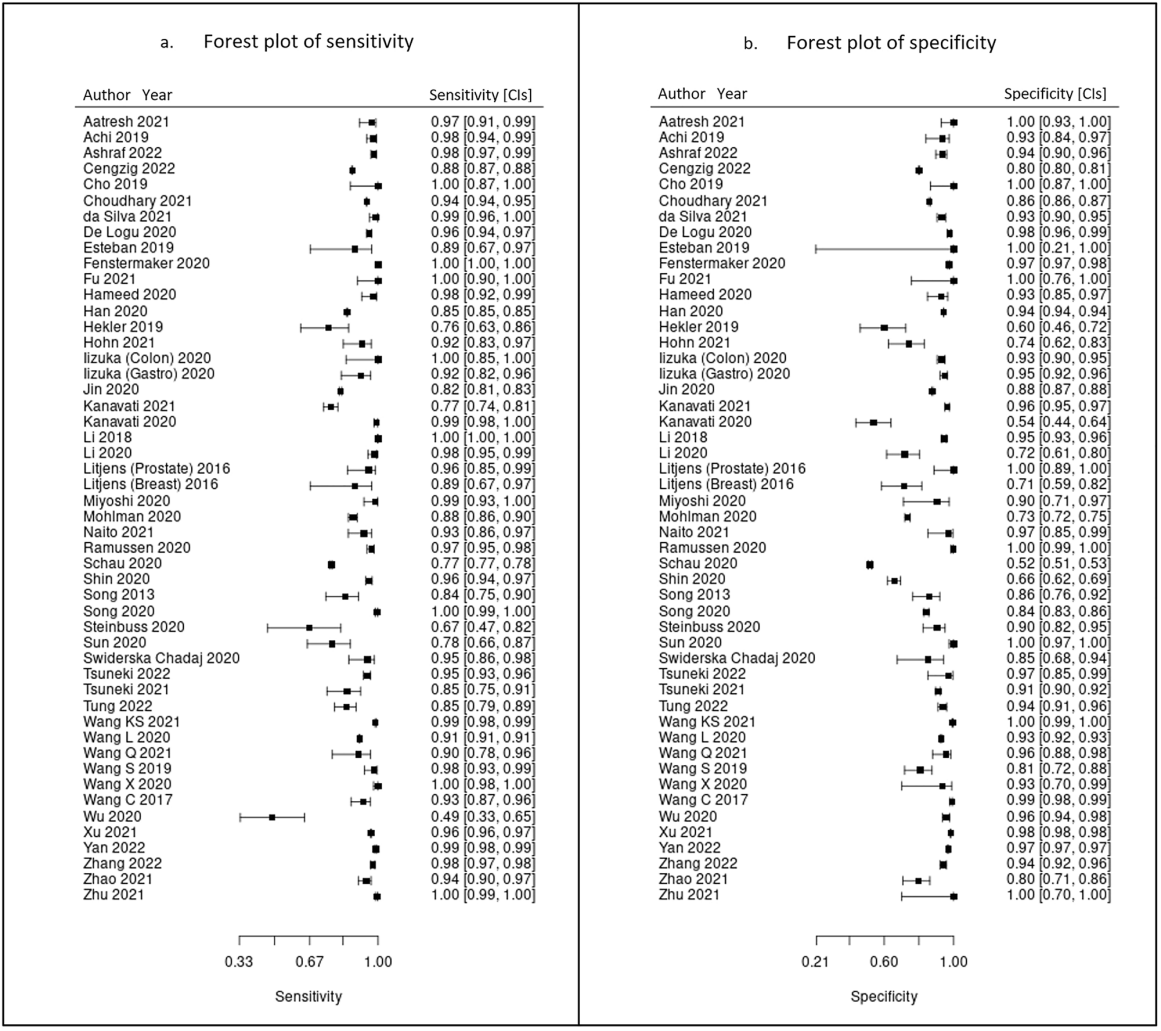
Forest plots of performance across studies included in the meta-analysis. These show sensitivity (**a**) and specificity (**b**) in studies of all pathologies with 95% confidence intervals. These plots were generated by MetaDTA: Diagnostic Test Accuracy Meta-Analysis v2.01 Shiny App https://crsu.shinyapps.io/MetaDTA/ and the raw data can be found in Supplementary Table 4^92,93^.

**Fig. 5:**
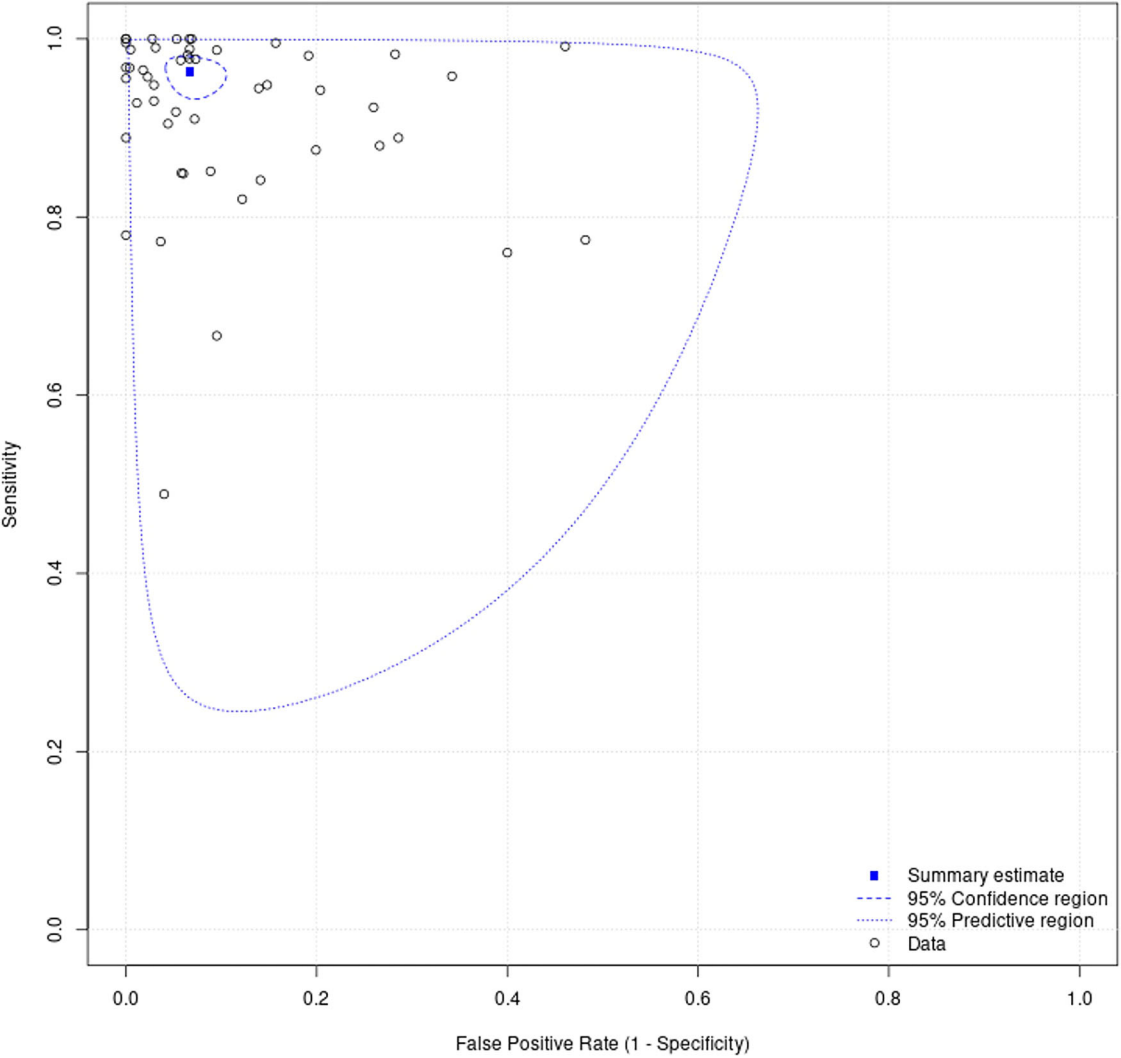
Summary receiver operating characteristic plot of AI applied to whole slide images for all disease types generated from MetaDTA: diagnostic test accuracy meta-analysis v2.01 Shiny App https://crsu.shinyapps.io/dta_ma/^92,93^. 95% confidence intervals are shown around the summary estimate. The predictive region shows the area of 95% confidence in which the true sensitivity and specificity of future studies lies, whilst factoring the statistical heterogeneity of studies demonstrated in this review.

100 studies were identified for inclusion in this systematic review. Included study size varied greatly from 4 WSIs to nearly 30,000 WSIs. Data on a WSI level was frequently unavailable for numbers used in test sets, but where it was reported this ranged from 10 WSI to nearly 14,000 WSIs, with a mean of 822 WSIs and a median of 113 WSIs. The majority of studies had small datasets and just a few studies contained comparatively large datasets of thousands or tens of thousands of WSIs. Of included studies, 48 had data that could be meta-analysed. Two of the studies in the meta-analysis had available data for two different disease types^27,28^, meaning a total of 50 assessments included in the meta-analysis. Figure 4 shows the forest plots for sensitivity of any AI solution applied to whole slide images. Overall, there was high diagnostic accuracy across studies and disease types. Using a bivariate random effects model, the estimate of mean sensitivity across all studies was 96.3% (CI 94.1–97.7) and of mean specificity was 93.3% (CI 90.5–95.4), as shown in Fig. 5. Additionally, the F1 score was calculated for each study (Supplementary Materials) from the raw confusion matrix data and this ranged from 0.43 to 1, with a mean F1 score of 0.87. Raw data and additional data for the meta-analysis can be found in Supplementary Tables 3 and 4.

The largest subgroups of studies available for inclusion in the meta-analysis were studies of gastrointestinal pathology^28–40^, breast pathology^27,41–47^ and urological pathology^27,48–54^ which are shown in Table 8, representing over 60% of models included in the meta-analysis. Notably, studies of gastrointestinal pathology had a mean sensitivity of 93% and mean specificity of 94%. Similarly, studies of uropathology had mean sensitivities and specificities of 95% and 96% respectively. Studies of breast pathology had slightly lower performance at mean sensitivity of 83% and mean specificity of 88%. Results for all other disease types are also included in the meta-analysis^55–74^. Forest plots for these subgroups are shown in Supplementary figure 1. When examining cancer (48 of 50 models) versus for non-cancer diseases (2 of 50 models), performance was better for the former with mean sensitivity 92% and mean specificity 89% compared to mean sensitivity of 76% and mean specificity of 88% respectively. For studies that could not be included in the meta-analysis, an indication of best performance from other accuracy metrics provided is outlined in Supplementary Table 2.

**Table 8:** Mean performance across studies by pathological subspecialty

Pathological subspecialty	No. AI models	Mean sensitivity	Mean specificity
Gastrointestinal pathology	14	93%	94%
Breast pathology	8	83%	88%
Uropathology	8	95%	96%
Hepatobiliary pathology	5	90%	87%
Dermatopathology	4	89%	81%
Cardiothoracic pathology	3	98%	76%
Haematopathology	3	95%	86%
Gynaecological pathology	2	87%	83%
Soft tissue & bone pathology	1	98%	94%
Head & neck pathology	1	98%	72%
Neuropathology	1	100%	95%

Of models examined in the meta-analysis, the number of sources ranged from one to fourteen and overall the mean sensitivity and specificity improved with a larger number of data sources included in the study. For example, mean sensitivity and specificity for one data source was 89% and 88% respectively, whereas for three data sources this was 93% and 92% respectively. However, the majority of studies used one or two data sources only, meaning that studies with larger numbers of data sources were comparably underrepresented. Additionally, of these models, the mean sensitivity and specificity was higher in those validated on an external test set (95% and 92% respectively compared to those without external validation (91% and 87% respectively), although it must be acknowledged that frequently raw data was only available for internal validation performance. Similar performance was reported across studies that had a slide-level and patch/tile-level unit of analysis with a mean sensitivity of 95% and 91% respectively versus a mean specificity of 88% and 90% respectively. When comparing tasks where data was provided in a multiclass confusion matrix compared to a binary confusion matrix, multiclass tasks demonstrated slightly better performance with a mean sensitivity of 95% and mean specificity of 92% compared to binary tasks with mean sensitivity 91% and mean specificity 88%. Details of these analyses can be found in Supplementary Tables 5–9.

Of papers included within the meta-analysis, details of specimen preparation were frequently not specified, despite this potentially impacting the quality of histopathological assessment and subsequent AI performance. In addition, the majority of models in the meta-analysis used haematoxylin and eosin (H&E) images only, with two models using H&E combined with IHC, making comparison of these two techniques difficult. Further details of these findings can be found in Supplementary Table 11.

## Discussion

AI has been extensively promoted as a useful tool that will transform medicine, with examples of innovation in clinical imaging, electronic health records (EHR), clinical decision making, genomics, wearables, drug development and robotics^75–80^. The potential of AI in digital pathology has been identified by many groups, with discoveries frequently emerging and attracting considerable interest^9,81^. Tools have not only been developed for diagnosis and prognostication, but also for predicting treatment response and genetic mutations from the H&E image alone^8,9,11^. Various models have now received regulatory approval for applications in pathology, with some examples being trialled in clinical settings^54,82^.

Despite the many interesting discoveries in pathology AI, translation to routine clinical use remains rare and there are many questions and challenges around the evidence quality, risk of bias and robustness of the medical AI tools in general^22–24,83,84^. This systematic review and meta-analysis addresses the diagnostic accuracy of AI models for detecting disease in digital pathology across all disease areas. It is a broad review of the performance of pathology AI, addresses the risk of bias in these studies, highlights the current gaps in evidence and also the deficiencies in reporting of research. Whilst the authors are not aware of a comparable systematic review and meta-analysis in pathology AI, Aggarwal et al. performed a similar review of deep learning in other (non-pathology) medical imaging types and found high diagnostic accuracy in ophthalmology imaging, respiratory imaging and breast imaging^75^. Whilst there are many exciting developments across medical imaging AI, ensuring that products are accurate and underpinned by robust evidence is essential for their future clinical utility and patient safety.

### Findings

This study sought to determine the diagnostic test accuracy of artificial intelligence solutions applied to whole slide images to diagnose disease. Overall, the meta-analysis showed that AI has a high sensitivity and specificity for diagnostic tasks across a variety of disease types in whole slide images (Figs. 4 and 5). The F1 score (Supplementary Materials) was variable across the individual models included in the meta-analysis. However, on average there was good performance demonstrated by the mean F1 score. The performance of the models described in studies that were not included in the meta-analysis were also promising (see Supplementary Materials).

Subgroups of gastrointestinal pathology, breast pathology and urological pathology studies were examined in more detail, as these were the largest subsets of studies identified (see Table 8 and Supplementary Materials). The gastrointestinal subgroup demonstrated high mean sensitivity and specificity and included AI models for colorectal cancer^28–30,32,34,40^, gastric cancer^28,31,33,37–39,85^ and gastritis^35^. The breast subgroup included only AI models for breast cancer applications, with Hameed et al. and Wang et al. demonstrating particularly high sensitivity (98%, 91% respectively) and specificity (93%, 96% respectively)^42,45^. However, there was lower diagnostic accuracy in the breast group compared to some other specialties. This could be due to several factors, including challenges with tasks in breast cancer itself, an over-estimation of performance and bias in other areas and the differences in datasets and selection of data between subspecialty areas. Overall results were most favourable for the subgroup of urological studies with both high mean sensitivity and specificity (Table 8). This subgroup included models for renal cancer^48,52^ and prostate cancer^27,49–51,53,54^. Whilst high diagnostic accuracy was seen in other subspecialties (Table 8), for example mean sensitivity and specificity in neuropathology (100%, 95% respectively) and soft tissue and bone pathology (98%, 94% respectively), there were very few studies in these subgroups and so the larger subgroups are likely more representative.

Of studies of other disease types included in the meta-analysis (Fig. 4), AI models in liver cancer^74^, lymphoma^73^, melanoma^72^, pancreatic cancer^71^, brain cancer^67^ lung cancer^57^ and rhabdomyosarcoma^56^ all demonstrated a high sensitivity and specificity. This emphasises the breadth of potential diagnostic tools for clinical applications with a high diagnostic accuracy in digital pathology. The majority of studies did not report details of the fixation and preparation of specimens used in the dataset. Where frozen section is used instead of formalin fixed paraffin embedded (FFPE) samples, this could impact the digital image quality and impact AI performance. It would be helpful for authors to consider including this information in the methods section of future studies. Only two models included in the meta-analysis used IHC and this was in combination with H&E stained samples. It would be interesting to explore the comparison between tasks using H&E when compared to IHC in more detail in future work.

Sensitivity and specificity were higher in studies with a greater number of included data sources, however few studies chose to include more than two sources of data. To develop AI models that can be applied in different institutions and populations, a diverse dataset is an important consideration for those conducting research into models intended for clinical use. A higher mean sensitivity and specificity for those models that included an external validation was identified, although many studies did not include this, or included most data for internal validation performance. Improved overall reporting of these values would allow a greater understanding of the performance of models at external validation. Performance was similar in the models included in the meta-analysis when a slide-level or patch/tile-level analysis was performed, although slide-level performance could be more useful when interpreting the clinical implications of a proposed model. A pathologist will review a case for diagnosis at slide level, rather than patch level, and so slide-level performance may be more informative when considering use in routine clinical practice. Performance was lower in non-cancer diseases when compared to cancer models, however only two of the models included in the meta-analysis were for non-cancer diseases and so this must be interpreted with caution and further work is needed in these disease areas.

Risk of bias and applicability assessments highlighted that the majority of papers contained at least one area of concern, with many studies having multiple areas of concern (Fig. 3 and Supplementary Materials). Poor reporting of the pieces of essential information within the studies was an issue that was identified at multiple points within this review. This was a key factor in the risk of bias and applicability assessment, as frequently important information that was either missing or ambiguous in its description. Reporting guidelines such as CLAIM and also STARD-AI (currently in development) are useful resources that could help authors to improve the completeness of reporting within their studies^29^^,^^86^. Greater endorsement and awareness of these guidelines could help to improve the completeness of reporting of this essential information in a study. The consequence of identifying so many studies with areas of concern, means that if the work were to be replicated with these concerns addressed, there is a risk that a lower diagnostic accuracy performance would be found. For this review, with 98–99% of studies containing areas of concern, any results for diagnostic accuracy need to be interpreted with caution. This is concerning due to the risk of undermining confidence of the use of AI tools if real world performance is poorer than expected. In future, greater transparency and reporting of the details of datasets, index test, reference standard and other areas highlighted could help to ameliorate these issues.

### Limitations

It must be acknowledged that there is uncertainty in the interpretation of the diagnostic accuracy of the AI models demonstrated in these studies. There was substantial heterogeneity in the study design, metrics used to demonstrate diagnostic accuracy, size of datasets, unit of analysis (e.g. slide, patch, pixel, specimen) and the level of detail given on the process and conduct of the studies. For instance, the total number of WSIs used in the studies for development and testing of AI models ranged from less than ten WSIs to tens of thousands of WSIs^87,88^. As discussed, of the 100 papers identified for inclusion in this review, 99% had at least one area at high or uncertain risk of bias or applicability concerns and similarly of the 48 papers included in the meta-analysis, 98% had at least one area at risk. Results for diagnostic accuracy in this paper should therefore be interpreted with caution.

Whilst 100 papers were identified, only 48 studies were included in the meta-analysis due to deficient reporting. Whilst the meta-analysis provided a useful indication of diagnostic accuracy across disease areas, data for true positive, false positive, false negative and true negative was frequently missing and therefore made the assessment more challenging. To address this problem, missing data was requested from authors. Where a multiclass study output was provided, this was combined into a 2 × 2 confusion matrix to reflect disease detection/diagnosis, however this offers a more limited indication of diagnostic accuracy. AI specific reporting guidelines for diagnostic accuracy should help to improve this problem in future^86^.

Diagnostic accuracy in many of the described studies was high. There is likely a risk of publication bias in the studies examined, with studies of similar models with lower reported performance on testing that are likely missing from the literature. AI research is especially at risk of this, given it is currently a fast moving and competitive area. Many studies either used datasets that were not randomly selection or representative of the general patient population, or were unclear in their description of case selection, meaning studies were at risk of selection bias. The majority of studies used either one or two data sources only and therefore the training and test datasets may have been comparatively similar. All of these factors should be considered when interpreting performance.

## Conclusions

There are many promising applications for AI models in WSIs to assist the pathologist. This systematic review has outlined a high diagnostic accuracy for AI across multiple disease types. A larger body of evidence is available for gastrointestinal pathology, urological pathology and breast pathology. Many other disease areas are underrepresented and should be explored further in future. To improve the quality of future studies, reporting of sensitivity, specificity and raw data (true positives, false positives, false negatives, true negatives) for pathology AI models would help with transparency in comparing diagnostic performance between studies. Providing a clear outline of the breakdown of data and the data sources used in model development and testing would improve interpretation of results and transparency. Performing an external validation on data from an alternative source to that on which an AI model was trained, providing details on the process for case selection and using large, diverse datasets would help to reduce the risk of bias of these studies. Overall, better quality study design, transparency, reporting quality and addressing substantial areas of bias is needed to improve the evidence quality in pathology AI and to therefore harness the benefits of AI for patients and clinicians.

## Methods

This systematic review and meta-analysis was conducted in accordance with the guidelines for the “Preferred Reporting Items for Systematic Reviews and Meta-Analyses” extension for diagnostic accuracy studies (PRISMA-DTA)^89^. The protocol for this review is available https://www.crd.york.ac.uk/prospero/display_record.php?ID = CRD42022341864 (Registration: CRD42022341864).

### Eligibility criteria

Studies reporting the diagnostic accuracy of AI models applied to WSIs for any disease diagnosed through histopathological assessment and/or immunohistochemistry (IHC) were sought. This included both formalin fixed tissue and frozen sections. The primary outcome was the diagnostic accuracy of AI tools in detecting disease or classifying subtypes of disease. The index test was any AI model applied to WSIs. The reference standard was any diagnostic histopathological interpretation by a pathologist and/or immunohistochemistry.

Studies were excluded where the outcome was a prediction of patient outcomes, treatment response, molecular status, whilst having no detection or classification of disease. Studies of cytology, autopsy and forensics cases were excluded. Studies grading, staging or scoring disease, but without results for detection of disease or classification of disease subtypes were also excluded. Studies examining modalities other than whole slide imaging or studies where WSIs were mixed with other imaging formats were also excluded. Studies examining other techniques such as immunofluorescence were excluded.

### Data sources and search strategy

Electronic searches of PubMed, EMBASE and CENTRAL were performed from inception to 20th June 2022. Searches were restricted to English language and human studies. There were no restrictions on the date of publication. The full search strategy is available in Supplementary Note 1. Citation checking was also conducted.

### Study selection

Two investigators (C.M. and H.F.A.) independently screened titles and abstracts against a predefined algorithm to select studies for full text review. The screening tool is available in Supplementary Note 2. Disagreement regarding study inclusion was resolved by discussion with a third investigator (D.T.). Full text articles were reviewed by two investigators (C.M. and E.L.C.) to determine studies for final inclusion.

### Data extraction and quality assessment

Data collection for each study was performed independently by two reviewers using a predefined electronic data extraction spreadsheet. Every study was reviewed by the first investigator (C.M.) and a team of four investigators were used for second independent review (E.L.C./C.J./G.M./C.C.). Data extraction obtained the study demographics; disease examined; pathological subspecialty; type of AI; type of reference standard; datasets details; split into train/validate/test sets and test statistics to construct 2 × 2 tables of the number of true-positives (TP), false positives (FP), false negatives (FN) and true negatives (TN). An indication of best performance with any diagnostic accuracy metric provided was recorded for all studies. Corresponding authors of the primary research were contacted to obtain missing performance data for inclusion in the meta-analysis.

At the time of writing, the QUADAS-AI tool was still in development and so could not be utilised^90^. Therefore, a tailored QUADAS-2 tool was used to assess the risk of bias and any applicability concerns for the included studies^86,91^. Further details of the quality assessment process can be found in Supplementary Note 3.

### Statistical analysis

Data analysis was performed using MetaDTA: Diagnostic Test Accuracy Meta-Analysis v2.01 Shiny App to generate forest plots, summary receiver operating characteristic (SROC) plots and summary sensitivities and specificities, using a bivariate random effects model^92,93^. If available, 2 × 2 tables were used to include studies in the meta-analysis to provide an indication of diagnostic accuracy demonstrated in the study. Where unavailable, this data was requested from authors or calculated from other metrics provided. For multiclass tasks where only multiclass data was available, the data was combined into a 2 × 2 confusion matrix (positives and negatives) format to allow inclusion in the meta-analysis. If negative results categories were unavailable for multiclass tasks, (e.g. for multiple comparisons between disease types only) then these had to be excluded. Additionally, mean sensitivity and specificity were examined in the largest pathological subspecialty groups, for cancer vs non-cancer diagnoses and for multiclass vs binary tasks to compare diagnostic accuracy among these studies.

### Supplementary information

Supplementary information

## Data Availability

All data generated or analysed during this study are included in this published article and its supplementary information files.
