# Supplementary material for: Artificial intelligence in digital pathology: a systematic review and meta-analysis of diagnostic test accuracy
Source: NPJ Digit Med. 2024 May 4;7:114. doi: 10.1038/s41746-024-01106-8 (PMC11069583; doi:10.1038/s41746-024-01106-8)
Supplement: Supplementary file 1 — Supplementary information [file 41746_2024_1106_MOESM1_ESM.pdf]

Supplementary Information

Contents

SUPPLEMENTARY NOTES ... 2

Supplementary note 1 – Search strategy of three databases (PubMed, EMBASE & CENTRAL) ... 2

Supplementary note 2 – Screening tools for inclusion of articles ... 3

Supplementary note 2a: Screening tool for abstracts ... 3

Supplementary note 2b: Screening tool for full text articles ... 3

Supplementary note 3 – QUADAS-2 tool tailored for this review ... 4

SUPPLEMENTARY FIGURES ... 5

Supplementary figure 1 – Supplementary forest plots of sensitivity and specificity for subgroups ... 5

Supplementary figure 1a – Forest plots for sensitivity and specificity in studies of gastrointestinal pathology ... 5

Supplementary figure 1b – Forest plots for sensitivity and specificity in studies of breast pathology ... 5

Supplementary figure 1c – Forest plots for sensitivity and specificity in studies of urological pathology ... 6

Supplementary figure 1d – Forest plots for sensitivity and specificity in studies of other pathologies ... 6

SUPPLEMENTARY TABLES ... 7

Supplementary table 1 – Individual paper scores for QUADAS-2 assessment* ... 7

Supplementary table 2 – Other accuracy / performance metrics for papers not included in the meta-analysis ... 9

Supplementary table 3 – Meta analysis: additional data & source of data ... 10

Supplementary table 4 – Raw data for forest plots Figure 4 (main text) ... 11

Supplementary table 5 – Performance by number of included data sources in the meta-analysis ... 12

Supplementary table 6 – Performance of models including an external validation in the meta-analysis ... 12

Supplementary table 7 – Performance of models by unit of analysis in the meta-analysis ... 13

Supplementary table 8 – Performance of models by task type (binary / multiclass) in the meta-analysis ... 13

Supplementary table 9 – Performance of models by disease type (cancer / non cancer) in the meta-analysis ... 13

Supplementary table 10 – Further details of study characteristics for all included studies ... 14

Supplementary table 11 – Breakdown of sample staining and preparation for papers included in the meta-analysis ... 19

Supplementary table 11a – Use of haematoxylin & eosin (H&E) compared to immunohistochemistry (IHC) for models included in the meta-analysis ... 19

Supplementary table 11b – Sample preparation and fixation for cases used to develop and test models included in the meta-analysis ... 19

SUPPLEMENTARY NOTES

Supplementary note 1 – Search strategy of three databases (PubMed, EMBASE & CENTRAL)

Pubmed
Limits (Humans, English)

1	digital pathol*.ti,ab.	961
2	whole slide image.ti,ab.	176
3	histopathol*.ti,ab.	132,723
4	artificial intelligence.ti,ab.	12,011
5	deep learning.ti,ab.	14,760
6	machine learning.ti,ab.	31,756
7	neural network.ti,ab.	21,974
8	computer vision.ti,ab.	2,141
9	support vector machine.ti,ab.	8,911
10	#1 OR #2 OR #3	133,553
11	#4 OR #5 OR #6 OR #7 OR #8 OR #9	70,047
12	#10 AND #11	1,279

Embase Classic+Embase

1	digital pathol*.ti,ab.	1952
2	whole slide image.ti,ab.	408
3	histopathol*.ti,ab.	370,508
4	artificial intelligence.ti,ab.	23,908
5	deep learning.ti,ab.	31,614
6	machine learning.ti,ab.	67,418
7	neural network.ti,ab.	59,436
8	computer vision.ti,ab.	5,963
9	support vector machine.ti,ab.	19,875
10	1 or 2 or 3	372,380
11	4 or 5 or 6 or 7 or 8 or 9	166,702
12	10 and 11	2,628
13	limit 12 to (human and english language and (embase or medline))	1,537

CENTRAL

ID	Search Hits	
#1	"digital pathol*"	0
#2	"whole slide image"	14
#3	histopathol*	10,595
#4	"artificial intelligence"	1,141
#5	"deep learning"	729
#6	"machine learning"	1,904
#7	"neural network"	1,148
#8	"computer vision"	116
#9	"support vector machine"	376
#10	#1 OR #2 OR #3	10,603
#11	#4 OR #5 OR #6 OR #7 OR #8 OR #9	4,135
#12	#10 AND #11	180
#13	#12 in Trials	160

Supplementary note 2 – Screening tools for inclusion of articles

Supplementary note 2a: Screening tool for abstracts

1.	Is this article an original research paper?<br>(i.e. not a review, conference abstract, commentary etc.)	No = Reject<br>Yes = Next question
2.	Is education the primary focus of the article?	Yes = Reject<br>No = Next question
3.	Is this article examining whole slide imaging?<br>(i.e. not other imaging modalities e.g. other pathology imaging technologies, radiological imaging, endoscopy etc.)	No = Reject<br>Yes = Next question
4.	Is this article examining a surgical pathology / histopathology problem(s)? (i.e. not cytology, autopsy, toxicology, forensics, descriptions of new systems or collaborations)	No = Reject<br>Yes = Next question
5.	Is this article examining artificial intelligence for whole slide imaging?<br>(i.e. not manual annotation etc.)	No = Reject<br>Yes = Next question
6.	Is this study examining diagnosis of a disease?<br>(I.e. not determining only prognosis, treatment response, molecular status etc or focused on a purely quality / technical issue for WSI))	No = Reject<br>Yes = Next question
7.	Is this study measuring diagnostic accuracy?<br>(i.e. referring to accuracy or including accuracy statistics)	No = Reject<br>Yes = Next question
8.	Is this a study of humans?<br>(i.e. not an animal based study)	No = Reject<br>Yes = Next question
9.	Is this study written in English?	No = Reject<br>Yes = Accept

Supplementary note 2b: Screening tool for full text articles

1.	Is this article an original research paper?	No = Reject<br>Yes = Next question
2.	Is education the primary focus of the article?	Yes = Reject<br>No = Next question
3.	Is this article examining whole slide imaging?<br>(not other modalities and not combined with other modalities in the analysis)	No = Reject<br>Yes = Next question
4.	Is this article examining a surgical pathology / histopathology problem(s)?	No = Reject<br>Yes = Next question
5.	Is this article examining artificial intelligence for whole slide imaging?	No = Reject<br>Yes = Next question
6.	Is this study examining diagnosis of a disease?<br>(detection of disease or classification of disease subtypes only)	No = Reject<br>Yes = Next question
7.	Is this study measuring diagnostic accuracy?	No = Reject<br>Yes = Next question
8.	Is this a study of humans?	No = Reject<br>Yes = Next question
9.	Is this study written in English?	No = Reject<br>Yes = Accept
10.	Does the ground truth use or imply use of human pathologist using H&E or IHC?	No = Reject<br>Yes = Accept
11.	Does the article describe a grand challenge exercise with models from multiple authors?<br>(Rather than diagnostic accuracy study from one group)	Yes = Reject<br>No = Accept

Supplementary note 3 – QUADAS-2 tool tailored for this review

Appendix – Adapted QUADAS2 tool

Domain 1: Patient Selection

Risk of Bias (describe methods of patient selection)

Signaling questions

- Was a consecutive or random sample of cases used in the test set(s)? Yes/No/Unclear
- Did the study avoid inappropriate exclusions? (I.e. excluding all artefacts or excluding cases that were difficult to diagnose) Yes/No/Unclear

QUESTION 1 - Could the selection of patients have introduced bias? RISK: LOW/HIGH/UNCLEAR

Low risk (1) if all the answers to signalling questions were 'yes'
High risk (2) if any of the answers to signalling questions were 'no'
Unclear (3) if answer to signalling questions was 'unclear'

Concerns regarding applicability (describe included patients)

QUESTION 2 – Is there a concern that the included patients do not match the review question? CONCERN: LOW/HIGH/UNCLEAR

Low risk (1) if cases were selected from a given condition, without excluding subgroups
High risk (2) if subgroups of cases with a given condition were excluded, not reflecting the full case mix
Unclear (3) if it is not clear how cases were selected

Domain 2: Index Test(s)

Risk of bias (describe the index test and how it was conducted and interpreted)

Signaling questions

- Were the reported performance results from test data that was independent of the training data? Yes/No/Unclear
- Was the index test tested on an external independent test set? Yes/No/Unclear
- Was the same image analysis performed on all the cases? Yes/No/Unclear
- Were all test cases used in the analysis? Yes/No/Unclear

QUESTION 3 – Could the conduct or interpretation of the index test have introduced bias? RISK: LOW/HIGH/UNCLEAR

Low risk (1) if all the answers to signalling questions were 'yes'
High risk (2) if any of the answers to signalling questions were 'no'
Unclear (3) if answer to signalling questions was 'unclear'

Concerns regarding applicability

QUESTION 4 – Is there a concern that the index test, its conduct, or interpretation differ from the review question? CONCERN: LOW/HIGH/UNCLEAR

Low risk (1) if there is no concern that the index test, its conduct or interpretation differ from the review question
High risk (2) if there is concern of either the index test, its conduct or interpretation differing from the review question
Unclear (3) if it is not clear if the index test, its conduct or interpretation differ from the review question.

Domain 3: Reference Standard

Risk of bias (describe the reference standard and how it was conducted and interpreted)

Signaling questions

- Is the reference standard likely to correctly classify the target condition? Yes/No/Unclear
- Were the reference standard results interpreted without knowledge of the results of the index test? Yes/No/Unclear

QUESTION 5 – Could the reference standard, its conduct, or its interpretation have introduced bias? RISK: LOW/HIGH/UNCLEAR

Low risk (1) if the answers to both signalling questions were 'yes'
High risk (2) if the answers to either signalling questions were 'no'
Unclear (3) if answer to either signalling questions was 'unclear'

Concerns regarding applicability

QUESTION 6 – Is there concern that the target condition as defined by the reference standard does not match the review question? CONCERN: LOW/HIGH/UNCLEAR

Low risk (1) if the criteria for diagnosis was clearly defined and the target condition diagnosed by a pathologist.
High risk (2) if the criteria for diagnosis was not clearly defined or if the target condition was not diagnosed by a pathologist.
Unclear (3) if the criteria for diagnosis of a given condition was unclear or if it is not clear who diagnosed the target condition.

Domain 4: Flow and Timing

Risk of bias (describe the index test and how it was conducted and interpreted)

Signaling questions

1. Was the time interval between diagnosis of the reference standard and the scanning of the glass slides for whole slide images <10 years? Yes/No/Unclear

QUESTION 7 – Could the case flow have have introduced bias? RISK: LOW/HIGH/UNCLEAR

Low risk (1) if answer to signalling question was 'yes'
High risk (2) if answer to signalling question was 'no'
Unclear (3) if answer to signalling question was 'unclear'

SUPPLEMENTARY FIGURES

Supplementary figure 1 – Supplementary forest plots of sensitivity and specificity for subgroups

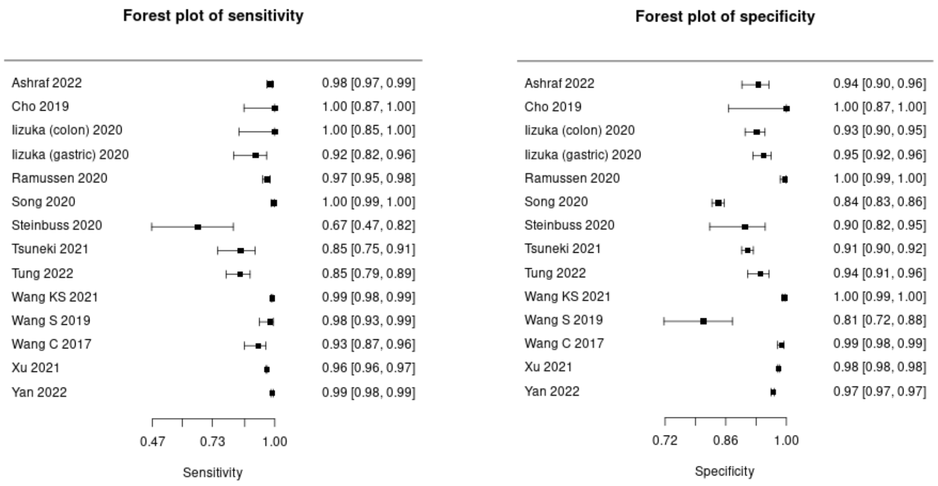

Supplementary figure 1a – Forest plots for sensitivity and specificity in studies of gastrointestinal pathology

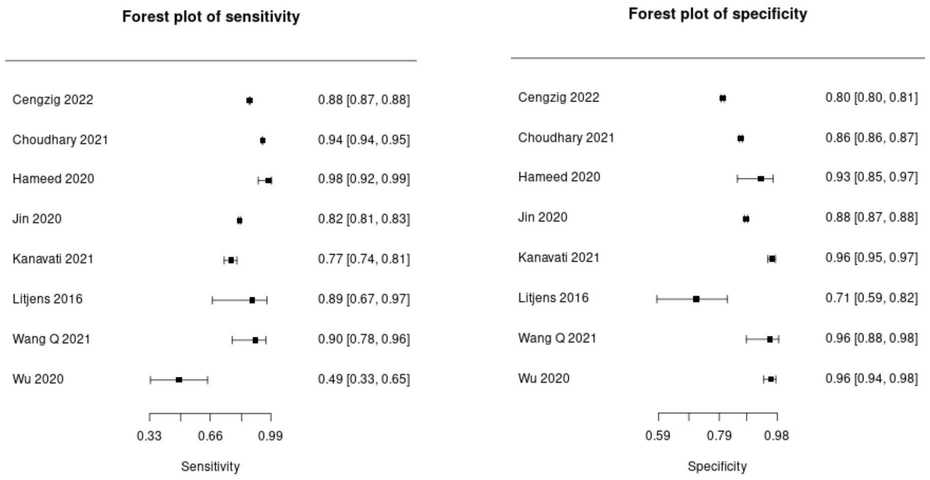

Supplementary figure 1b – Forest plots for sensitivity and specificity in studies of breast pathology

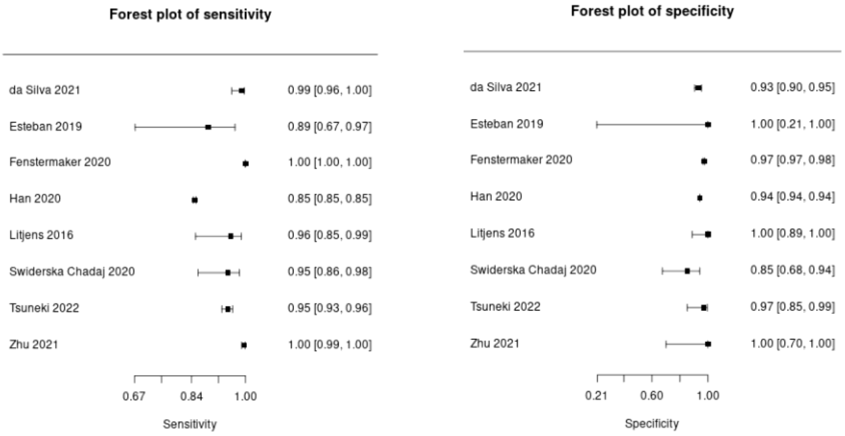

Supplementary figure 1c – Forest plots for sensitivity and specificity in studies of urological pathology

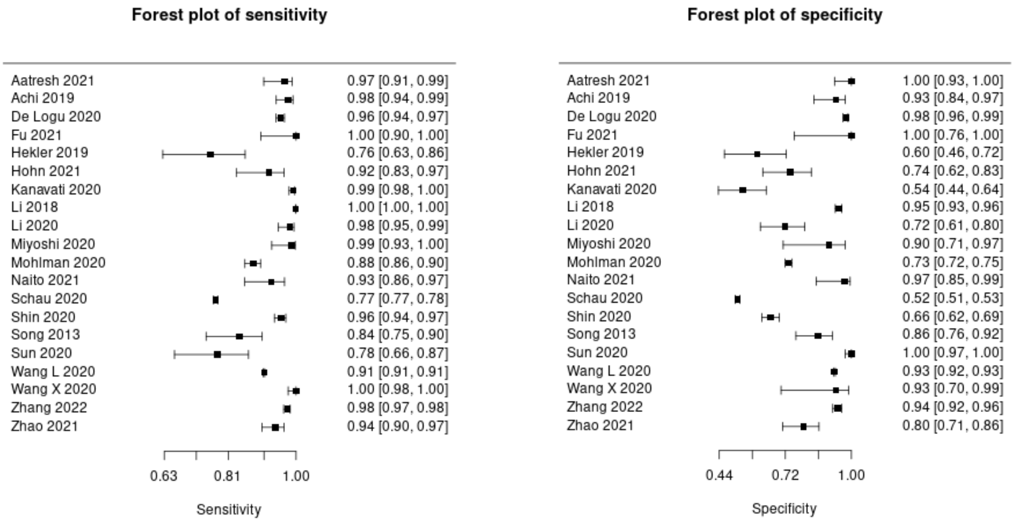

Supplementary figure 1d – Forest plots for sensitivity and specificity in studies of other pathologies

SUPPLEMENTARY TABLES

Supplementary table 1 – Individual paper scores for QUADAS-2 assessment*

First author	Publication year	Risk of Bias				Concerns of Applicability		
		Patient selection	Index test	Reference standard	Flow and timing	Patient selection	Index test	Reference standard
Aatresh[74]	2021	3	2	3	3	3	1	3
Abdeltawab[131]	2021	1	2	1	3	3	1	1
Achi[73]	2019	2	2	3	3	3	3	3
Alheejawi[86]	2021	3	2	3	1	3	3	3
Ashraf[39]	2022	2	2	1	3	3	1	1
Ba[127]		3	2	1	3	3	1	1
BenTaieb[133]	2017	3	1	1	3	3	1	1
Cengiz[47]	2022	3	3	3	3	3	3	3
Chen[103]	2021	3	1	1	1	3	1	1
Chen[114]	2020	2	1	1	3	3	1	3
Chen[104]	2022	3	2	1	3	3	3	1
Cho[38]	2019	2	3	1	3	3	3	3
Choudhary[46]	2021	2	2	1	3	3	3	3
Coudray[105]	2018	2	2	1	3	3	1	1
Cruz-Roa[94]	2018	3	1	1	3	3	1	3
Cruz-Roa[95]	2017	3	1	1	3	3	1	3
da Silva[54]	2021	1	2	1	1	1	1	1
De Logu[72]	2020	3	2	1	3	3	3	1
Dehkharghanian[106]	2021	2	1	3	3	3	1	1
del Amor[113]	2021	3	2	1	3	3	1	1
DiPalma[139]	2021	3	2	3	3	3	1	2
Duran-Lopez[128]	2021	3	2	3	3	3	3	3
Esteban[53]	2019	2	1	1	3	3	1	3
Feng[121]	2021	2	2	3	3	3	1	2
Fenstermaker[52]	2020	2	2	3	3	3	3	1
Fu[71]	2021	2	1	3	3	3	1	3
Hameed[45]	2020	3	2	3	3	3	1	2
Han[129]	2020a	3	2	1	3	3	1	1
Han[51]	2020b	3	2	1	3	3	1	1
Haryanto[122]	2021	2	2	3	3	3	2	2
Hekler[70]	2019	1	2	1	1	1	1	1
Hohn[69]	2021	3	2	1	3	3	3	1
Huang[130]	2021	1	2	1	2	1	1	1
Iizuka[28]	2020	3	1	1	3	3	1	1
Jin[44]	2020	2	2	3	3	3	1	2
Johny[96]	2021	2	2	3	3	3	1	2
Kanavati[68]	2020	3	1	1	3	3	1	1
Kanavati[43]	2021	3	1	1	3	3	1	1
Khalil[97]	2022	3	2	1	3	3	1	3
Kiani[115]	2020	1	1	1	1	1	1	2
Kimeswenger[111]	2020	3	2	1	3	3	1	1
Li[112]	2021	3	2	1	3	3	1	3
Li[67]	2018	3	2	3	3	3	1	2
Li[66]	2020	3	2	1	1	3	1	1
Lin[98]	2019	2	2	3	3	3	1	2
Litjens[27]	2016	1	2	1	1	1	1	1
Ma[126]	2020	3	2	1	3	3	1	2
Menon[140]	2022	2	2	3	3	3	3	3
Mishra[142]	2017	3	3	1	3	3	3	2
Miyoshi[65]	2020	3	2	1	1	3	2	1
Mohlman[64]	2020	3	2	1	3	3	1	1
Naito[63]	2021	3	2	1	1	3	1	1
Nasir-Moin[119]	2021	2	1	1	1	3	1	1
Noorbakhsh[87]	2020	2	2	3	3	3	1	3
Rasmussen[37]	2020	1	1	1	3	1	1	2
Roy[99]	2021	2	2	3	3	3	3	3
Sabol[123]	2020	2	3	1	3	3	3	2
Sadeghi[100]	2019	2	2	1	3	3	3	2
Sali[117]	2020	3	2	3	1	3	1	3
Schau[62]	2020	3	2	1	3	3	1	1
Schilling[141]	2018	3	2	3	1	3	1	3
Schrammen[124]	2022	3	3	3	3	2	3	3
Shin[61]	2020	2	3	1	3	3	1	2
Song[60]	2013	3	2	3	3	3	3	3
Song[36]	2020a	3	1	3	3	3	1	3
Song[85]	2020b	1	1	1	1	1	1	1
Steinbuss[35]	2020	3	2	1	3	3	1	1
Steiner[101]	2018	3	1	1	1	3	1	1
Sun[59]	2020	3	2	1	1	3	3	1
Swiderska-Chadaj[50]	2020	3	1	1	3	3	1	2
Syed[118]	2021	3	3	1	2	3	1	1
Syrykh[135]	2020	3	2	1	3	3	1	1
Tabibu[132]	2019	2	2	3	3	3	1	3

Tsuneki[34]	2021	2	2	1	3	2	1	1
Tsuneki[49]	2022	1	1	1	3	1	1	1
Tung[33]	2022	2	2	1	1	3	1	3
Uegami[110]	2022	1	2	1	1	1	1	1
Valkonen[102]	2017	3	2	3	3	3	3	3
Wang KS[40]	2021	3	1	1	3	3	1	3
Wang L[58]	2020	3	1	1	2	3	1	1
Wang Q[42]	2021	2	1	3	3	3	1	3
Wang S[31]	2019	3	2	3	3	3	1	3
Wang X[57]	2020	2	1	3	3	3	1	3
Wang C[32]	2017	2	2	3	3	3	3	3
Wei[120]	2020	3	1	1	1	1	1	1
Wei[107]	2019	3	2	1	1	3	1	1
Wu[41]	2020	3	3	3	3	3	3	3
Xu[138]	2017	2	2	1	3	3	1	3
Xu[30]	2021	2	1	1	3	2	1	3
Yan[29]	2022	2	3	3	3	3	3	3
Yang[108]	2021	3	1	1	3	3	1	1
Yang[116]	2022	3	3	1	3	3	3	2
Yu[134]	2020a	2	2	1	3	3	1	1
Yu[136]	2020b	2	3	1	3	3	3	1
Yu[137]	2021	3	3	1	3	3	1	1
Zhang[56]	2022	3	3	1	1	2	2	1
Zhao[55]	2021	2	2	3	3	3	1	2
Zheng[109]	2022	3	2	3	3	3	2	2
Zhou[125]	2021	3	1	1	3	3	3	1
Zhu[48]	2021	3	1	1	3	3	1	1

*Key

Colours within the table are representative of the level of risk scored using the adapted QUADAS-2 tool outlined in S3.

Risk of bias:

1 = Low risk
2 = High risk
3 = Unclear risk

Risk of applicability concerns:

1 = Low risk
2 = High risk
3 = Unclear risk

Supplementary table 2 – Other accuracy / performance metrics for papers not included in the meta-analysis

First author	Publication year	Reported performance (indication of best performance where multiple sets of results)
Abdeltawab[131]	2021	Average accuracy 0.957; sensitivity 0.920; specificity 0.971
Alheejawi[86]	2021	Accuracy 97.7%, precisions 83.22, recall 87.08%, dice 85.10, Jaccard 74.07
Ba[127]	2021	Overal accuracy 0.867. Best chronic atrophic gastritis: AUC 0.91, sens 0.952, spec 0.992, accuracy 0.986. Values given per disease class: Sensitivity 0.790-0.985; Specificity 0.829-1.000.
BenTaieb[133]	2017	Best accuracy Proposed model at 3 levels: 90.0%
Chen[103]	2021	(Best ADC & SCC) ADC AUC 0.9594 (0.9500-0.9689); SCC AUC 0.9414 (0.9243-0.9593)
Chen[114]	2020	(Detecting liver cancer) accuracy 0.960; Precision 0.945; Recall 1.000; F1 score 0.971. 89.6% accuracy for grade prediction
Chen[104]	2022	AUC 0.984 (per slide accuracy tumour detection WIFPS); accuracy 0.903; sensitivity 0.868; specificity 0.946
Coudray[105]	2018	Normal vs tumour AUC 0.993 (0.974-1.0); 3 class at 5x best AUC 0.981 (0.968-0.980)
Cruz-Roa[94]	2018	Dice 0.76 +/- 0.20; PPV 0.72 +/- 0.22; NPV 0.97 +/- 0.05. (TPR 87%, TNR 92%, FPR 8%, FNR 13
Cruz-Roa[95]	2017	Dice 0.7586 +/- 0.2006; PPV 0.7162 +/- 0.2204; NPV 0.9677 +/- 0.0511
Dehkharghanian[106]	2021	Precision 0.92; Recall 0.91; F1 score 0.91 (average), accuracy 0.86-0.91
del Amor[113]	2021	Sensitivity 0.9285; Specificity 0.9202; PPV 0.8622; NPV 0.9599; F1 score 0.8942; Accuracy 0.9231; AUC 0.9244.
DiPalma[139]	2021	KD (ADv2 model) - Coeliac: Accuracy 87.2, F1 score 75.86, Precision 76.46, Recall 78.0; KD model - Lung: Accuracy 94.18, F1 score 79.63, Precision 79.75, Recall 82.0; KD model - Renal: Accuracy 89.11, F1 Score 77.1, Precision 75.66, Recall 82.64.
Duran-Lopez[128]	2021	Accuracy 94.24%; Sensitivity 98.87%; Precision 90.23%; F1 score 94.33%; AUC 0.94
Feng[121]	2021	Segmentation task: DSC 77.89%, Classification task: AUC 100%
Han[129]	2020	AlexNet-TCM: AUROC 0.964; error rate 6.1%; FNR 15.1%; FPR 5.8%
Haryanto[122]	2021	Best model taken as 300px+50px overlap. For image classification as malignant: Warwick dataset: sensitivity: 0.69; specificity: 0.93. UI dataset: sensitivity: 0.98; specificity: 1
Huang[130]	2021	Distinguishing cancer from benign epithelium & stroma: AUROC=0.92 (95%CI 0.88-0.95);<br>Cancer detection: weighted k = 0.97 (95%CI 0.96-0.98);<br>Cancer grading: weighted k = 0.98 (95%CI 0.96-1)
Johny[96]	2021	Accuracy 0.9184; Precision 0.9185; Recall 0.9183; F1 score 0.9183; AUC 0.97 (triangular model)
Khalil[97]	2022	Precision 0.892; Recall 0.837; F1 score 0.844; mIoU 0.749
Kiani[115]	2020	Accuracy 0.885 (0.710-0.960) (CNN alone on internal set); Accuracy 0.842 (0.808-0.876) (CNN alone on external set)
Kimeswenger[111]	2020	Accuracy 0.95; F1 score 0.97; AUC 0.99; Sensitivity 0.96; Specificity 0.93.
Li[112]	2021	AUC 0.971
Lin[98]	2019	FROC (tumour localisation): 0.8533; AUROC (classification): 0.9875.
Ma[126]	2020	AUC 0.9876; accuracy 96%; specificity 93.3%; sensitivity 98.7%
Menon[140]	2022	Accuracy: BRCA 0.97, COAD 0.99, KICH 0.98, KIRP 0.95, LIHC 0.98; LUAD 0.95, LUSC 0.95, PRAD 0.92, READ 0.97, STAD 0.96
Mishra[142]	2017	Accuracy 0.924; Precision 0.97; Recall 0.94; F1-score 0.95
Nasir-Moin[119]	2021	Accuracy model + pathologist best: 80.8% (78.8-82.8)
Noorbakhsh[87]	2020	All tumour types (19) slide level: AUC 0.995 (+/- 0.008). All tumours types tile based: accuracy 0.91 (+/- 0.05); precision 0.97 (+/- 0.02); recall 0.90 (+/- 0.06); specificity 0.86 (+/- 0.07)
Roy[99]	2021	Accuracy 0.922; Precision 0.931; Recall 0.887; F1 score 0.908.
Sabol[123]	2020	CNN Balanced: Accuracy 92.74%; Precision 92.5%; Recall 92.76%; F1 92.64%
Sadeghi[100]	2019	97.8% accuracy on validation set. On testing the 25% quantile of the probability score of the predictions increased from 0.48 to 0.89, and the median of the data increased from 0.95 to 0.99.
Sali[117]	2020	Best model GMM-RF: Average - accuracy 0.952 (0.915-0.989); AUC 0.986 (0.970-1.000); Precision 0.9555 (0.930-0.980); Recall 0.941 (0.903-0.979); F1 score 0.942 (0.904-0.981)
Schilling[141]	2018	Sensitivity 87.5%; Specificity 80%; PPV 83%; F1 score 88.9%; NPV 100%
Schrammen[124]	2022	AUROC 0.980 (0.975, 0.984) (on training set)
Song[36]	2020	Accuracy 90.4%; AUC 0.92;
Steiner[101]	2018	Sensitivity 91.2% (86-96.5%) P=0.023 (assisted read across images on case basis); AUC 98.5-0.99
Syed[118]	2021	Multi-zoom ResNet50 patch level (same CM): Macro AUC 0.95; Accuracy 95% at patch level, sensitivity 0.96, specificity 0.97, PPV 0.96, NPV 0.97, Precision 0.94, Recall 0.94, F1 score 0.94. Modified ReNet50 with the ensemble: AUC 0.99, Accuracy 98.3%, Sensitivity 95%, Specificity 96%. Multi-zoom ResNet50 biopsy level: AUC 0.99; accuracy 0.98; sensitivity 0.96; Specificity 0.97; PPV 0.96; NPV 0.97; Precision 0.94; Recall 0.94.
Syrykh[135]	2020	AUC 0.99, accuracy 91%
Tabibu[132]	2019	ResNet-18 (KIRC) Cancer v Normal: patch wise accuracy 93.39; Precision 93.41; Recall 92.95; Slide wise AUC 0.99.
Uegami[110]	2022	Test set: Best AUC 0.88 (0.78-0.98). Sensitivity 0.89; Specificity 0.74.
Valkonen[102]	2017	Training: Accuracy 93%; Sensitivity 92.6%; Specificity 93.3%; F-score 0.93. Best AUC 0.98464 (0.97995 - 0.98932) cross validation. Random Forest sensitivity 92.6%, specificity 93.3%, F-score 0.93.
Wei[120]	2020	Internal mean: accuracy 93.5%; sensitivity 86.8%; specificity 95.7%. External mean: accuracy 87.0%; sensitivity 77.7%; specificity 91.6%
Wei[107]	2019	Kappa score 0.525; average agreement 66.6%; robust agreement 76.7%
Xu[138]	2017	Brain cancer classification (best): Accuracy 97.8%. Segmenting: accuracy 84%. CRC binary best: accuracy 98.0%. CRC multiclass 87.2%.
Yang[108]	2021	EfficientNetB5 on SYSU1 (best): Macro average AUC 0.988 (0.982-0.994); accuracy 0.860; weighted F1 score 0.860
Yang[116]	2022	(FA-MSCN 5x_2.5x) Sensitivity 0.96; Intersection over union (IOU) 0.89
Yu[134]	2020a	AUC 0.975 (+/- 0.001) (Tumour detection)
Yu[136]	2020b	AUC 0.985 (+/- 0.004) (SCC vs benign); AUC 0.971 (+/- 0.007) (AdenoCa vs benign)
Yu[137]	2021	AUC 0.996 (CI 0.949-0.984) (case level but 1 slide per case)
Zheng[109]	2022	TCGA ext test set normal v tumour: AUC 0.980 (+/- 0.04). 3 label task TCGA: Average accuracy 82.3; average AUC 92.8.
Zhou[125]	2021	Combination framework: Accuracy 0.946, Precision 0.964, Recall 0.982, F1 score 0.973

Note: This data was obtained for performance from test sets. Occasionally, detail about the breakdown of model development and tests sets was vague or absent. In these cases, the authors of this review have endeavoured to obtain what appears to be the test set results from the paper.

Supplementary table 3 – Meta analysis: additional data & source of data

Author	TP	FN	FP	TN	N	F1 score
Aatresh 2021[74]	90	3	0	50	143	0.98
Achi 2019[73]	176	4	4	56	240	0.98
Ashraf 2022[39]	485	9	16	231	741	0.97
Cengiz 2022[47]	63322	9029	5848	23507	101706	0.89
Cho 2019[38]	25	0	0	25	50	1.00
Choudhary 2021[46]	56333	3311	3289	20325	83258	0.94
da Silva 2021[54]	173	2	27	377	579	0.92
De Logu 2020[72]	1074	48	18	773	1913	0.97
Esteban 2019[53]	16	2	0	1	19	0.94
Fenstermaker 2020[52]	12046	0	85	3000	15131	1.00
Fu 2021[71]	35	0	0	12	47	1.00
Hameed 2020[45]	86	2	6	76	170	0.96
Han 2020[51]	32092	5689	70530	1140166	1248477	0.46
Hekler 2019[70]	38	12	20	30	100	0.70
Hohn 2021*[69]	60	5	17.4	49.6	132	0.84
Iizuka (Colon) 2020[28]	21	0	33	446	500	0.56
Iizuka (Gastro) 2020[28]	56	5	23	416	500	0.80
Jin 2020[44]	13435	2949	1999	14385	32768	0.84
Kanavati 2020[68]	586	5	41	48	680	0.96
Kanavati 2021[43]	431	127	30	794	1382	0.85
Li 2018[67]	6944	2	56	998	8000	1.00
Li 2020[66]	171	3	24	61	259	0.93
Litjens (Prostate) 2016[27]	43	2	0	30	75	0.98
Litjens (Breast) 2016[27]	16	2	16	40	74	0.64
Miyoshi 2020[65]	78	1	2	19	100	0.98
Mohlman 2020[64]	741	101	860	2372	4074	0.61
Naito 2021[63]	80	6	1	33	120	0.96
Rasmussen 2020[37]	446	15	2	508	971	0.98
Schau 2020[62]	16250	4737	4862	5228	31077	0.77
Shin 2020[61]	594	26	212	408	1240	0.83
Song 2013[60]	69	13	11	67	160	0.85
Song 2020[85]	630	3	405	2174	3212	0.76
Steinbuss 2020[35]	16	8	8	76	108	0.67
Sun 2020[59]	46	13	0	141	200	0.88
Swiderska Chadaj 2020[50]	55	3	4	23	85	0.94
Tsuneki 2021[34]	63	11	153	1572	1799	0.43
Tsuneki 2022[49]	695	38	1	33	767	0.97
Tung 2022[33]	157	28	22	343	550	0.86
Wang KS 2021[40]	3940	48	9	1842	5839	0.99
Wang L 2020[58]	60289	5963	1215	15660	83127	0.94
Wang Q 2021[42]	38	4	3	65	110	0.92
Wang S 2019[31]	104	2	18	76	200	0.91
Wang X 2020[57]	170	0	1	14	185	1.00
Wang C 2017[32]	116	9	10	865	1000	0.92
Wu 2020*[41]	17.6	18.4	15.7	376.3	428	0.51
Xu 2021[30]	19300	700	360	19640	40000	0.97
Yan 2022[29]	1397	14	267	8322	10000	0.91
Zhang 2022[56]	1056	26	34	558	1674	0.97
Zhao 2021[55]	213	13	21	82	329	0.93
Zhu 2021[48]	904	4	0	9	917	1.00

*Data provided by authors as averages of a cross validation (not whole numbers)

Colour key for source of meta-analysis data	
	Retrieved from study / supplementary materials
	Multiclass confusion matrix in study reduced to 2x2 table
	Back-calculated from data provided in study
	Provided by author
	Back-calculated from data provided by author
	Multiclass confusion matrix provided by author reduced to 2x2 table

Supplementary table 4 – Raw data for forest plots Figure 4 (main text)

Author	Sensitivity	Lower 95% CI	Upper 95% CI
Aatresh 2021[74]	0.97	0.91	0.99
Achi 2019[73]	0.98	0.94	0.99
Ashraf 2022[39]	0.98	0.97	0.99
Cengiz 2022[47]	0.88	0.87	0.88
Cho 2019[38]	1.00	0.87	1.00
Choudhary 2021[46]	0.94	0.94	0.95
da Silva 2021[54]	0.99	0.96	1.00
De Logu 2020[72]	0.96	0.94	0.97
Esteban 2019[53]	0.89	0.67	0.97
Fenstermaker 2020[52]	1.00	1.00	1.00
Fu 2021[71]	1.00	0.90	1.00
Hameed 2020[45]	0.98	0.92	0.99
Han 2020[51]	0.85	0.85	0.85
Hekler 2019[70]	0.76	0.63	0.86
Hohn 2021[69]	0.92	0.83	0.97
Iizuka (Colon) 2020[28]	1.00	0.85	1.00
Iizuka (Gastric) 2020[28]	0.92	0.82	0.96
Jin 2020[44]	0.82	0.81	0.83
Kanavati 2020[68]	0.99	0.98	1.00
Kanavati 2021[43]	0.77	0.74	0.81
Li 2018[67]	1.00	1.00	1.00
Li 2020[66]	0.98	0.95	0.99
Litjens (Breast) 2016[27]	0.89	0.67	0.97
Litjens (Prostate) 2016[27]	0.96	0.85	0.99
Miyoshi 2020[65]	0.99	0.93	1.00
Mohlman 2020[64]	0.88	0.86	0.90
Naito 2021[63]	0.93	0.86	0.97
Ramussen 2020[37]	0.97	0.95	0.98
Schau 2020[62]	0.77	0.77	0.78
Shin 2020[61]	0.96	0.94	0.97
Song 2013[60]	0.84	0.75	0.90
Song 2020[85]	1.00	0.99	1.00
Steinbuss 2020[35]	0.67	0.47	0.82
Sun 2020[59]	0.78	0.66	0.87
Swiderska Chadaj 2020[50]	0.95	0.86	0.98
Tsuneki 2021[34]	0.85	0.75	0.91
Tsuneki 2022[49]	0.95	0.93	0.96
Tung 2022[33]	0.85	0.79	0.89
Wang C 2017[32]	0.93	0.87	0.96
Wang KS 2021[40]	0.99	0.98	0.99
Wang L 2019[58]	0.91	0.91	0.91
Wang Q 2021[42]	0.90	0.78	0.96
Wang S 2019[31]	0.98	0.93	0.99
Wang X 2020[57]	1.00	0.98	1.00
Wu 2020[41]	0.49	0.33	0.65
Xu 2021[30]	0.96	0.96	0.97
Yan 2022[29]	0.99	0.98	0.99
Zhang 2022[56]	0.98	0.97	0.98
Zhao 2021[55]	0.94	0.90	0.97
Zhu 2021[48]	1.00	1.00	1.00

Author	Specificity	Lower 95% CI	Upper 95% CI
Aatresh 2021[74]	1.00	0.93	1.00
Achi 2019[73]	0.93	0.84	0.97
Ashraf 2022[39]	0.94	0.90	0.96
Cengiz 2022[47]	0.80	0.80	0.81
Cho 2019[38]	1.00	0.87	1.00
Choudhary 2021[46]	0.86	0.86	0.87
da Silva 2021[54]	0.93	0.90	0.95
De Logu 2020[72]	0.98	0.96	0.99
Esteban 2019[53]	1.00	0.21	1.00
Fenstermaker 2020[52]	0.97	0.97	0.98
Fu 2021[71]	1.00	0.76	1.00
Hameed 2020[45]	0.93	0.85	0.97
Han 2020[51]	0.94	0.94	0.94
Hekler 2019[70]	0.60	0.46	0.72
Hohn 2021[69]	0.74	0.62	0.83
Iizuka (Colon) 2020[28]	0.93	0.90	0.95
Iizuka (Gastric) 2020[28]	0.95	0.92	0.96
Jin 2020[44]	0.88	0.87	0.88
Kanavati 2020[68]	0.54	0.44	0.64
Kanavati 2021[43]	0.96	0.95	0.97
Li 2018[67]	0.95	0.93	0.96
Li 2020[66]	0.72	0.61	0.80
Litjens (Breast) 2016[27]	0.71	0.59	0.82
Litjens (Prostate) 2016[27]	1.00	0.89	1.00
Miyoshi 2020[65]	0.91	0.71	0.97
Mohlman 2020[64]	0.73	0.72	0.75
Naito 2021[63]	0.97	0.85	0.99
Rasmussen 2020[37]	1.00	0.99	1.00
Schau 2020[62]	0.52	0.51	0.53
Shin 2020[61]	0.66	0.62	0.69
Song 2013[60]	0.86	0.76	0.92
Song 2020[85]	0.84	0.83	0.86
Steinbuss 2020[35]	0.90	0.82	0.95
Sun 2020[59]	1.00	0.97	1.00
Swiderska Chadaj 2020[50]	0.85	0.68	0.94
Tsuneki 2021[34]	0.91	0.90	0.92
Tsuneki 2022[49]	0.97	0.85	0.99
Tung 2022[33]	0.94	0.91	0.96
Wang C 2017[32]	0.99	0.98	0.99
Wang KS 2021[40]	1.00	0.99	1.00
Wang L 2019[58]	0.93	0.92	0.93
Wang Q 2021[42]	0.96	0.88	0.98
Wang S 2019[31]	0.81	0.72	0.88
Wang X 2020[57]	0.93	0.70	0.99
Wu 2020[41]	0.96	0.94	0.98
Xu 2021[30]	0.98	0.98	0.98
Yan 2022[29]	0.97	0.97	0.97
Zhang 2022[56]	0.94	0.92	0.96
Zhao 2021[55]	0.80	0.71	0.86
Zhu 2021[48]	1.00	0.70	1.00

Supplementary table 5 – Performance by number of included data sources in the meta-analysis

No. data sources	No. models	Mean sensitivity (%)	Mean specificity (%)
1	23	89%	88%
2	18	95%	92%
3	4	93%	92%
4	1	99%	54%
5	1	85%	91%
6	1	95%	97%
14	1	99%	100%
Not stated	1	88%	80%

Supplementary table 6 – Performance of models including an external validation in the meta-analysis

External validation of the model	No. models	Mean sensitivity (%)	Mean specificity (%)
No	26	91%	87%
Unclear	3	78%	90%
Yes	21	95%	92%

Supplementary table 7 – Performance of models by unit of analysis in the meta-analysis

Unit of analysis	No. models	Mean sensitivity (%)	Mean specificity (%)
Other	2	74%	95%
Patch / Tile	28	91%	90%
Slide	20	95%	88%

Supplementary table 8 – Performance of models by task type (binary / multiclass) in the meta-analysis

Task type	No. models	Mean sensitivity (%)	Mean specificity (%)
Binary	39	91%	88%
Multiclass	11	95%	92%

Supplementary table 9 – Performance of models by disease type (cancer / non cancer) in the meta-analysis

Disease type	No. models	Mean sensitivity (%)	Mean specificity (%)
Cancer	48	92%	89%
Non-cancer	2	76%	88%

Supplementary table 10 – Further details of study characteristics for all included studies

First author	Publication year	Funding source of research	Intended use*	Pathological subspecialty/ies	Total number of slides in study (other units if not provided)	Number of data sources	Is the dataset(s) open source	Is the test set independent of the training set
Aatresh[74]	2021	Science Engineering and Research Board, Department of Science and Technology, Govt. of India	Classifying subtypes of liver cancer	Hepatobiliary pathology	398 WSI (141 WSI into 705 patches for TCGA), (257 WSI into 1338 patches for KMC)	2	Mixed	Unclear
Abdeltawab[131]	2021	No funder declared	Classifying subtypes of renal cancer	Uropathology	64 WSIs	1	No	Yes
Achi[73]	2019	No funder declared	Classifying lymphoma subtypes	Haematopathology	128 WSIs (equalling 2560 40x40 pixel patches)	2	Unclear	Yes
Alheejawi[86]	2021	Natural Sciences and Engineering Research Council of Canada; Ministry of Higher Education and Scientific Research, Iraq; Imam Ja'afar Al Sadiq University, Iraq	Detecting melanoma	Dermatopathology	4 WSIs	1	No	Yes
Ashraf[39]	2022	Seegene Medical Foundation, South Korea	Detecting gastric cancer	Gastrointestinal pathology	905 WSIs and 327,680 96x96 pixel patches	2	Mixed	Yes
Ba[127]	2021	PLA General Hospital Medical Big Data and Artificial Intelligence Project	Classifying subtypes of gastritis	Gastrointestinal pathology	1250 WSIs	1	No	Yes
BenTaieb[133]	2017	Natural Sciences and Engineering Research Council of Canada	Classifying subtypes of ovarian cancer	Gynaecological pathology	133 WSIs	1	Yes	Yes
Cengiz[47]	2022	No funder declared	Detecting breast cancer	Breast pathology	398,381 50x50 size patches	Not stated	Unclear	Unclear
Chen[103]	2021	Ministry of Sciences and Technology Taiwan	Classifying subtypes of lung cancer	Cardiothoracic pathology	7003 WSIs hospitals set; 1044 WSIs TCGA test set.	4	Mixed	Yes
Chen[114]	2020	Opening Fund of Engineering Research Center of Cognitive Healthcare of Zhejiang Province, Zhejiang Medical Health Science and Technology Project, National Natural Science Foundation of China	Detecting liver cancer; grading liver cancer severity	Hepatobiliary pathology	592 WSIs	2	Mixed	Yes
Chen[104]	2022	National Key R&D program of China; National Natural Science Foundation of China; Guangdong Natural Science Foundation.	Detecting lung cancer, classifying subtype of lung cancer	Cardiothoracic pathology	1914 cases	3	No	Yes
Cho[38]	2019	National Research Foundation of Korea; Catholic Medical Centre Research Foundation	Detecting gastric cancer	Gastrointestinal pathology	803 WSIs	2	Mixed	Yes
Choudhary[46]	2021	No funder declared	Detecting breast cancer	Breast pathology	162 WSIs	1	Yes	Yes
Coudray[105]	2018	Cancer Centre Support Grant, Laura and Isaac Perlmutter Cancer Centre.	Detecting lung cancer & classification of non-small cell lung cancer subtypes	Cardiothoracic pathology	1634 WSIs (TCGA) + 340 WSIs (New York) independent set	2	Mixed	Yes
Cruz-Roa[94]	2018	Administrative Department of Science, Technology and Innovation - Colciencias, Universidad Nacional de Colombia; Universidad de los Llanos; the National Cancer Institute of the National Institutes of Health; National Institute of Diabetes and Digestive and Kidney Diseases; National Center for Research Resources; United States Department of Defense Prostate Cancer Synergistic Idea Development Award; United States Department of Defense Lung Cancer Idea Development New Investigator Award; United States Department of Defense Prostate Cancer Idea Development Award; United States Department of Defense Peer Reviewed Cancer Research Program Case Comprehensive Cancer Center Pilot Grant; VelaSano Grant, Cleveland Clinic; the Wallace H. Coulter Foundation Program Case Western Reserve University.	Detecting breast cancer	Breast pathology	945 cases	4	Mixed	Yes
Cruz-Roa[95]	2017	DGI-Unillanos; Administrative Department of Science, Technology and Innovation of Colombia; National Cancer Institutes of the National Institutes of Health; the National Institute of Diabetes and Digestive and Kidney diseases; National Center for Research Resources; DOD Prostate Cancer Synergistic Idea Development Award; DOD Lung Cancer Idea Development New Investigator Award; DOD Prostate Cancer Idea Development Award; DOD Peer Reviewed Cancer Research Program; Cleveland Clinic; Wallace H. Coulter Foundation Program, Case Western Reserve University.	Detecting breast cancer	Breast pathology	605 patients	4	Mixed	Yes
da Silva[54]	2021	Paige; Breast Cancer Research Foundation; National Institutes of Health / National Cancer Institute; P50 grant;	Detecting prostate cancer	Uropathology	661 WSIs (from 579 unique needle core biopsy parts	1	No	Yes
De Logu[72]	2020	Associazione Italiana per la Ricerca sul Cancro	Detecting melanoma	Dermatopathology	100 WSIs	3	No	Yes
Dehkharghanian[106]	2021	Government of Ontario, Canada and the Ontario Research Fund-Research Excellence Gigapixel image identification consortium	Classifying lung cancer subtypes	Cardiothoracic pathology	758 WSIs	2	Mixed	Yes
del Amor[113]	2021	Horizon 2020, the Spanish Ministry of Economy and Competitiveness, Instituto de Salud Carlos III, GVA, Polytechnic University of Valencia, Marie Skłodowska Curie grant	Detecting spitzoid melanocytic lesions	Dermatopathology	53 WSIs	1	No	Yes
DiPalma[139]	2021	US National Library of Medicine, US National Cancer Institute	Detecting coeliac disease; classifying lung cancer subtypes;	Multiple	Coeliac: 1364 patients; Lung: 269 WSIs;	2	Mixed	Yes

			classifying renal cancer subtypes		Renal 882 WSIs.				
Duran-Lopez[128]	2021	Spanish Agencia Estatal de Investigation, European Regional Development Fund	Detecting prostate cancer	Uropathology	332 WSIs	1	No	Unclear	
Esteban[53]	2019	Ministerio de Economía y Competitividad.	Detecting prostate cancer	Uropathology	79 WSIs from SICAPv1; and ext set 593 patches for testing from Gertych et al	1	Mixed	Yes	
Feng[121]	2021	National Key Research and Development Program of China; National Natural Science Foundation of China; Zhejiang University Education Foundation; Zhejiang public welfare technology research project; Key Laboratory of Medical Neurobiology of Zhejiang Province; NSF Grant.	Detecting colorectal cancer	Gastrointestinal pathology	1000 WSIs	1	Yes	Yes	
Fenstermaker[52]	2020	No funders declared	Detecting renal cell cancer. Classifying subtypes of RCC.	Uropathology	42 patients	1	Yes	Yes	
Fu[71]	2021	Foundation of Beijing Municipal Science and Technology Commission; National Key Research and Development Program of China; National Natural Science Foundation of China.	Detecting pancreatic ductal adenocarcinoma	Hepatobiliary pathology	283 WSIs	2	Mixed	Yes	
Hameed[45]	2020	Basque Country project MIFLUDAN; eVida Research Group IT 905-16 (University of Deusto, Spain)	Detecting breast cancer	Breast pathology	845 areas/patches from 544 WSIs.	1	No	Yes	
Han[129]	2020a	No funders declared	Detecting prostate cancer.	Uropathology	299 WSIs	1	No	Yes	
Han[51]	2020b	Canadian Institute of Health Research; Ontario Institute for Cancer Research; Prostate Canada; Natural Sciences and Engineering Research Council of Canada	Detecting prostate cancer	Uropathology	299 WSIs	1	No	Yes	
Haryanto[122]	2021	Ministry of Research and Technology, Republic of Indonesia	Detecting colorectal cancer	Gastrointestinal pathology	165 images + other images from University of Indonesia. (For best model (300px + 50px overlap), no. of CSW-generated images = 13,576 (2,984 (Warwick), 10,592 (UI))	2	Mixed	Unclear	
Hekler[70]	2019	No funders	Detecting melanoma	Dermatopathology	695 WSIs from 595 patients	1	No	Yes	
Hohn[69]	2021	Federal Ministry of Health, Berlin, Germany; Tumour Behaviour Prediction Initiative.	Detecting melanoma	Dermatopathology	431 WSIs	2	No	Yes	
Huang[130]	2021	PathomIQ	Detecting prostate cancer.	Uropathology	1000 WSIs	1	No	Yes	
Iizuka[28]	2020	No funders declared	Classifying gastric and colonic tumours	Gastrointestinal pathology	10,186 WSIs	2	Mixed	Yes	
Jin[44]	2020	CancerCare Manitoba Founation; Natural Sciences and Engineering Research Council of Canada; University of Manitoba; Manitoba Medical Services Foundation Allen Rouse Basic Science Career Development Research Award.	Detecting breast cancer metastases in lymph nodes	Breast pathology	327,680 patches (PCaM), 438 images (second dataset), 100 patches from 10 WSIs (Warwick)	3	Yes	Yes	
Johny[96]	2021	No funders declared	Detecting breast cancer metastases in lymph nodes	Breast pathology	327,680 patches from 400 WSIs	1	Yes	Yes	
Kanavati[43]	2021	No funders declared	Detecting breast cancer and DCIS	Breast pathology	3672 WSIs	2	No	Yes	
Kanavati[68]	2020	Research Institute for Information Technology, Kyushu University	Detecting lung cancer	Cardiothoracic pathology	5734 WSIs	4	Mixed	Yes	
Khalil[97]	2022	Ministry of Science and Technology of Taiwan	Detecting breast cancer metastases in lymph nodes	Breast pathology	188 WSIs (94 H&E, 94 matching IHC CK(AE1/AE3) WSIs)	1	No	Yes	
Kiani[115]	2020	Department of Pathology (Stanford University) Stanford Machine Learning Group and the Stanford Center for Artificial Intelligence in Medicine & Imaging	Classification of liver tumour subtypes	Hepatobiliary pathology	150 WSIs	2	Mixed	Yes	
Kimeswenger[111]	2020	ERC; REA; Promedica Stiftung; Swiss Cancer Research Foundation; Clinical Research Priority Program (CRPP), University of Zurich; Swiss National Science Foundation; European Academic of Dermatology and Venereology.	Detecting basal cell carcinoma	Dermatopathology	820 WSIs	2	No	Yes	
Li[112]	2021	The National Key Research and Development Program of China; Natural Science Foundation of China; Hunan Province Science Foundation; Changsha Muncipal Natural Science Foundation; Scientific Research Fund of Hunan Provincial Education Department.	Detecting melanoma	Dermatopathology	701 WSIs	2	Mixed	Yes	
Li[67]	2018	No funder declared	Classifying subtypes of brain tumour	Neuropathology	206 WSIs	1	No	Yes	
Li[66]	2020	No funder declared	Detecting thyroid cancer	Head & neck pathology	608 WSIs	1	No	Yes	
Lin[98]	2019	Hong Kong Innovation and Technology Commission; Hong Kong Research Grants Council; Global Partnership Fund, University of Warwick.	Detect breast cancer metastases in lymph nodes	Breast pathology	400 WSIs	1	Yes	Yes	
Litjens[27]	2016	StITPro Foundation	Detecting breast cancer metastases in sentinel lymph nodes & prostate cancer grading	Multiple	Prostate: 225 WSIs; Breast: 271 WSIs.	1	No	Yes	

Ma[126]	2020	Shanghai Science and Technology Committee; National Key R&D Program of China; National Natural Science Foundation of China; Cross-Institute Research Fund of Shanghai Jiao Tong University; Innovation Foundation of Translational Medicine of Shanghai Jiao Tong University School of Medicine; Technology Transfer Project of Science & Technology, Department of Shanghai Jiao Tong University School of Medicine	Detecting gastric cancer and classifying gastric disease	Gastrointestinal pathology	763 WSIs	1	No	Yes
Menon[140]	2022	Ihub-Data, International Institute of Information and Technology, Hyderabad	Detect multiple cancer types	Multiple	9792 WSIs	1	Yes	Yes
Mishra[142]	2017	Cancer Prevention and Research Institute of Texas (CPRIT)	Detecting osteosarcoma	Soft tissue & bone pathology	82 WSIs (64,000 patches)	Unclear	No	Yes
Miyoshi[65]	2020	Chugai Pharmaceutical Co. Ltd	Classify subtypes of Lymphoma	Haematopathology	388 sections	1	No	Yes
Mohlman[64]	2020	No funder declared	Classify subtypes of lymphoma	Haematopathology	10,818 patches from unknown no. slides (70 cases)	2	No	Yes
Naito[63]	2021	Research Institute for Information Technology Kyushu University	Detecting pancreatic ductal adenocarcinoma	Hepatobiliary pathology	532 WSIs	1	No	Yes
Nasir-Moin[119]	2021	National Cancer Institute; National Library of Medicine	Assisting the pathologist with classifying subtypes of colorectal polyp	Gastrointestinal pathology	846 WSIs used in experiment + 60 WSIs for other purposes	25	No	Yes
Noorbakhsh[87]	2020	NIH Cloud Credits Model Pilot, NIH Big Data to Knowledge (BD2K) program; Google Cloud; NCI grant.	Detecting multiple cancer types and subtype classification	Multiple	29,930 WSIs	2	Yes	Yes
Rasmussen[37]	2020	Nova Scotia Health Authority Research Fund	Detecting hereditary diffuse gastric cancer	Gastrointestinal pathology	17,636 patches	2	No	Yes
Roy[99]	2021	No funders	Detecting invasive ductal carcinoma of the breast	Breast pathology	162 WSIs; 277,524 patches	1	Yes	Unclear
Sabol[123]	2020	AI4EU project from European Union's Horizon 2020 research & innovation programme; Maria Currie RISE LIFEBOTS Exchange Grant; EU FlagEra Joint Progect Robocom++, 2017-2021	Detecting colorectal cancer	Gastrointestinal pathology	5000 tiles	1	Yes	Unclear
Sadeghi[100]	2019	BMBF grant	Detecting lymph node breast cancer metastases	Breast pathology	500 WSI (camelyon 17) + 20,000 patches (cameylon 16)	2	Yes	Yes
Sali[117]	2020	National Institute of Diabetes and Digestive and Kidney Diseases of the National Institutes of Health.	Detecting dysplastic barretts oesophagus and non-dysplastic barretts oesophagus	Gastrointestinal pathology	650 WSI	1	Unclear	Yes
Schau[62]	2020	National Cancer Institute; OHSU Center for Spatial Systems Biomedicine; Knight Diagnostic Laboratories; Biomedical Innovation Program Award, Oregon Clinical and Translational Research Institute.	Detecting liver metastasis and classifying origin site of liver metastases	Gastrointestinal pathology	285 WSIs	1	Unclear	Yes
Schilling[141]	2018	No funder declared	Detecting Hirsprungs disease	Paediatric pathology	307 WSIs	1	Yes	Yes
Schrammen[124]	2022	German Federal Ministry of Health; Max-Eder-Programme of the German Cancer Aid; NIHR; Yorkshire Cancer Research program; German Research Foundation; Interdisciplinary Research Program of the National Centre for Tumour Diseases, Germany; German Federal Ministry of Education and Research.	Detecting colorectal cancer	Gastrointestinal pathology	3337 cases	2	No	Yes
Shin[61]	2020	Ministry of Trade, Industry & Energy (Korea); Ministry of Health & Welfare (Korea)	Detecting ovarian cancer	Gynaecological pathology	10,296 patches, 174 patients + 58 cases for additional experiments	2	Mixed	Yes
Song[60]	2013	Basic Science Research Program, National Research Foundation of Korea, funded by the Ministry of Education, Science and Technology; INHA University Research Grant	Classifying types of pancreatic neoplasm	Hepatobiliary pathology	11 WSIs, 400 patches	1	No	Unclear
Song[36]	2020a	CAMS Innovation Fund for Medical Sciences; National Natural Science Foundation of China (NSFC); Tsinghua Initiative Research Programme.	Detecting colorectal adenomas	Gastrointestinal pathology	579 WSIs	3	No	Yes
Song[85]	2020b	National Natural Science Foundation of China; CAMS Innovation Fund for Medical Sciences; Medical Big Data and Artificial Intelligence Project of the Chinese PLA General Hospital; Tsinghua Initiative Research Program Grant; Beijing Hope Run Special Fund of Cancer Foundation of China.	Detecting gastric cancer	Gastrointestinal pathology	8153 WSIs	2	No	Yes
Steinbuss[35]	2020	No Funders	Classify subtypes of gastritis	Gastrointestinal pathology	1230 patches	1	No	Yes
Steiner[101]	2018	Google Brain Healthcare Technology Fellowship	Assist pathologist in detecting breast cancer metastases in lymph nodes	Breast pathology	339 WSIs	3	Mixed	Yes
Sun[59]	2020	National Basic Research Program of China; Science and Technology Major Project of Hubei Province (Next-Generation AI Technologies); Medical Science and Technology projects of China	Detecting endometrial cancer; classifying endometrial diseases	Gynaecological pathology	3502 patches	1	Mixed	Yes
Swiderska-Chadaj[50]	2020	Philips Digital and Computational Pathology	Detecting prostate cancer	Uropathology	717 WSIs	3	No	Yes
Syed[118]	2021	National Institute of Diabetes and Digestive and Kidney Diseases of the National Institutes of Health, Bill and Melinda Gates Foundation, University of Virginia Center for Engineering in Medicine, University of Virginia THRIV Scholar Career Development Award.	Detecting coeliac disease and environmental enteropathy	Gastrointestinal pathology	461 WSIs	3	No	Yes
Syrykh[135]	2020	No funder declared	Detecting follicular lymphoma	Haematopathology	491 WSIs (378 + 65 + 24 +24)	2	No	Yes

Tabibu[132]	2019	No funder declared	Detecting renal cancer and classifying subtype	Uropathology	2105 WSIs	1	Yes	Yes
Tsuneki[34]	2021	No funders	Detecting poorly differentiated colorectal cancer	Gastrointestinal pathology	2547 WSIs	5	No	Yes
Tsuneki[49]	2022	No funders	Detect prostate cancer	Uropathology	3694 WSIs	6	Mixed	Yes
Tung[33]	2022	No funders declared	Detecting gastric cancer	Gastrointestinal pathology	50 patients; 2750 image tiles.	1	Yes	Yes
Uegami[110]	2022	New Energy and Industrial Technology Development Organization (NEDO)	Detecting Usual Interstitial Pneumonia (UIP)	Cardiothoracic pathology	715 WSIs + 181 WSIs pretraining set	1	No	Yes
Valkonen[102]	2017	1. Academy of Finland<br>2. Tekes - The Finnish Funding Agency for Innovation<br>3. Cancer Society of Finland, Sigrid Juselius Foundation and Doctoral Programme of Computing and Electrical Engineering, Tampere University of Technology	Detecting breast cancer metastases in lymph nodes	Breast pathology	270 WSIs	1	Yes	Unclear
Wang KS[40]	2021	1. National Institutes of Health<br>2. Edward G. Schlieder Endowment and the Drs. W. C. Tsai and P. T. Kung Professorship in Biostatistics from Tulane University<br>3. National Key Research and Development Plan of China<br>4. National Natural Science Foundation of China<br>5. Jiangwang Educational Endowment.<br>6. Natural Science Foundation of Hunan Province	Detecting colorectal cancer	Gastrointestinal pathology	14,680 WSIs	14	Mixed	Yes
Wang L[58]	2020	National Natural Science Foundation of China	Detect eyelid melanoma	Dermatopathology	155 WSIs (83,126 patches)	2	No	Yes
Wang Q[42]	2021	National Natural Science Foundation of China, National KeyR&DProgram of China, KeyR&DProgram of Liaoning Province, Young and Middle-aged Talents Program of the National Civil Affairs Commission, Liaoning BaiQianWan Talents Program, University-Industry Collaborative Education Program.	Detecting breast cancer metastases in lymph nodes	Breast pathology	529 WSIs	2	Yes	Yes
Wang S[31]	2019	Hong Kong Innovation and Technology Commission; Shenzhen Science and Technology Program.	Classification of gastric cancer and dysplasia	Gastrointestinal pathology	608 WSIs	1	No	Yes
Wang X[57]	2020	Hong Kong Innovation and Technology Commission; National Natural Science Foundation of Chine; Shenzhen Science and Technology Program.	Classifying subtypes of lung cancer	Cardiothoracic pathology	1439 WSIs (939 WSI internal, 500 WSI external)	2	Mixed	Yes
Wang C[32]	2017	National Natural Science Foundation of China	Detecting colorectal cancer	Gastrointestinal pathology	10 WSIs (1000 150 x 150 pixel images)	1	Yes	Unclear
Wei[120]	2020	NIH; Geisel School of Medicine at Dartmouth; Norris Cotton Cancer Centre.	Classification of colorectal polyps	Gastrointestinal pathology	746 WSIs	2	No	Yes
Wei[107]	2019	No funders declared	Classification of lung adenocarcinoma histological patterns	Cardiothoracic pathology	422 WSIs	1	No	Yes
Wu[41]	2020	Information Technology for Cancer Research program and National Institutes of Health	Detecting breast cancer	Breast pathology	240 cases	1	No	Unclear
Xu[138]	2017	Microsoft Research; Beijing National Science Foundation in China; Technology and Innovation Commission of Shenzhen in China; Beijing Young Talent Project in China; Fundamental Research Funds for the Central Universities of China from the State Key Laboratory of Software Development Environment in Beihang University in China.	Detecting & classifying brain cancer. Detecting colorectal cancer	Multiple	brain 141 images, colon 717 cropped regions	2	Mixed	Yes
Xu[30]	2021	Guangzhou Key Medical Discipline Construction Project Fund; Guangzhou Science and Technology Plan Project; Guangdong Provincial Science and Technology Plan Project.	Detecting colorectal cancer	Gastrointestinal pathology	476 WSIs (263 + 218 -5 removed)	2	Mixed	Unclear
Yan[29]	2022	Science and Technology Innovation 2030-Key Project of China; Key-Area Research and Development Program of Guangdong Province, China.	Detecting colorectal cancer and colorectal polyps, detecting breast cancer lymph node metastases.	Multiple	NCT-CRC 100,000 patches. CAMELYON16 100,000 patches. In-house 20 patients.	3	Mixed	Unclear
Yang[108]	2021	National Key R&D Program of China; National Natural Science Foundation of China; Guangdong Natural Science Foundation; Support Scheme of Guangzhou for Leading Talents in Innovation and Entrepreneurship.	Classifying subtypes of lung cancer and other lung diseases	Cardiothoracic pathology	1693 WSIs	3	Mixed	Yes
Yang[116]	2022	Ministry of Science and Technology (MOST), Taiwan	Detecting hepatocellular carcinoma	Hepatobiliary pathology	46 WSIs	Unclear	Unclear	Yes
Yu[134]	2020a	Schlager Family Award for Digital Health Innovations; Partners' Innovation Discovery Grant; Blavatnik Centre for Computational Biomedicine Award; Harvard Data Science Fellowship.	Detecting serous ovarian carcinoma & predicting tumour grade	Gynaecological pathology	1375 WSIs	1	Yes	Yes
Yu[136]	2020b	National Cancer Institute; National Institutes of Health; National Human Genome Research Institute; National Institutes of Health; Mobilize Centre, Stanford University; Harvard Data Science Fellowship; Harvard Medical School Centre for Computational Biomedicine Award	Detecting lung cancer and classifying subtypes of lung cancer	Haematopathology		2	Yes	Yes
Yu[137]	2021	No funders	Detecting T cell lymphomas & classifying T cell lymphoma subtypes	Haematopathology	40 WSIs (1 per patient, 33 ROIs)	17	No	Yes
Zhang[56]	2022	Children's Cancer Fund of Dallas, the QuadW Foundation, the NIH grants NCI National Clinical Trials Network (NCTN) Operations Centre, NCTN SDC, Children's Oncology Group (COG) Biospecimen Bank, the Cancer Prevention and Research Institute of Texas.	Classifying subtypes of rhabdomyosarcoma	Soft tissue & bone pathology	272 WSIs	1	Unclear	Yes
Zhao[55]	2021	Major Research Plan of the National Natural Science Foundation of China, the Shanghai Hospital Development Centre Clinical Science and Technology Innovation project, the	Detecting lung cancer and classifying subtypes of lung cancer	Cardiothoracic pathology	2125 WSIs	1	Yes	Yes

		National Key R&D Program of China and the National Natural Science Foundation of China.						
Zheng[109]	2022	National Institutes of Health, Johnson & Johnson Enterprise Innovation Inc., American Heart Association, Karen Toffler Charitable Trust, National Science Foundation.	Detecting lung cancer and classifying lung cancer subtypes	Cardiothoracic pathology	4153 WSIs for train / validate / test + 665 WSIs used for earlier development	3	Yes	Yes
Zhou[125]	2021	Double-Class University project, the National Natural Science Foundation of China, and Postgraduate Research & Practice Innovation Program of Jiangsu Province	Detecting colorectal cancer	Gastrointestinal pathology	1396 WSIs	4	Mixed	Yes
Zhu[48]	2021	US National Library of Medicine; US National Cancer Institute	Classify renal tumour subtypes	Uropathology	1482 WSIs	2	Mixed	Yes

*Given the varied language used to describe intended use, these were broadly categorised into detecting disease or classifying subtypes of disease for those relevant to this study.

Supplementary table 11 – Breakdown of sample staining and preparation for papers included in the meta-analysis

Stain type	No. models n (%)
H&E only	48 (96)
IHC only	0 (0)
Combined H&E and IHC	2 (4)
Unclear	0 (0)

Supplementary table 11a – Use of haematoxylin & eosin (H&E) compared to immunohistochemistry (IHC) for models included in the meta-analysis

Sample preparation & fixation	No. models n (%)
FFPE only	8 (16)
Frozen section only	1 (2)
Both FFPE and Frozen cases	4 (8)
Unclear	37 (74)
Other	0 (0)

Supplementary table 11b – Sample preparation and fixation for cases used to develop and test models included in the meta-analysis
